# tBRD-1 Selectively Controls Gene Activity in the *Drosophila* Testis and Interacts with Two New Members of the Bromodomain and Extra-Terminal (BET) Family

**DOI:** 10.1371/journal.pone.0108267

**Published:** 2014-09-24

**Authors:** Ina Theofel, Marek Bartkuhn, Tim Hundertmark, Thomas Boettger, Stefanie M. K. Gärtner, Katja Leser, Stephan Awe, Michael Schipper, Renate Renkawitz-Pohl, Christina Rathke

**Affiliations:** 1 Philipps-University Marburg, Department of Biology, Marburg, Germany; 2 Institute for Genetics, Justus-Liebig-University, Giessen, Germany; 3 Department of Cardiac Development and Remodeling, Max-Planck-Institute for Heart and Lung Research, Bad Nauheim, Germany; Cardiff University, United Kingdom

## Abstract

Multicellular organisms have evolved specialized mechanisms to control transcription in a spatial and temporal manner. Gene activation is tightly linked to histone acetylation on lysine residues that can be recognized by bromodomains. Previously, the testis-specifically expressed bromodomain protein tBRD-1 was identified in *Drosophila*. Expression of tBRD-1 is restricted to highly transcriptionally active primary spermatocytes. tBRD-1 is essential for male fertility and proposed to act as a co-factor of testis-specific TATA box binding protein-associated factors (tTAFs) for testis-specific transcription. Here, we performed microarray analyses to compare the transcriptomes of *tbrd-1* mutant testes and wild-type testes. Our data confirmed that tBRD-1 controls gene activity in male germ cells. Additionally, comparing the transcriptomes of *tbrd-1* and tTAF mutant testes revealed a subset of common target genes. We also characterized two new members of the bromodomain and extra-terminal (BET) family, tBRD-2 and tBRD-3. In contrast to other members of the BET family in animals, both possess only a single bromodomain, a characteristic feature of plant BET family members. Immunohistology techniques not only revealed that tBRD-2 and tBRD-3 partially co-localize with tBRD-1 and tTAFs in primary spermatocytes, but also that their proper subcellular distribution was impaired in *tbrd-1* and tTAF mutant testes. Treating cultured male germ cells with inhibitors showed that localization of tBRD-2 and tBRD-3 depends on the acetylation status within primary spermatocytes. Yeast two-hybrid assays and co-immunoprecipitations using fly testes protein extracts demonstrated that tBRD-1 is able to form homodimers as well as heterodimers with tBRD-2, tBRD-3, and tTAFs. These data reveal for the first time the existence of single bromodomain BET proteins in animals, as well as evidence for a complex containing tBRDs and tTAFs that regulates transcription of a subset of genes with relevance for spermiogenesis.

## Introduction

During spermatogenesis male germ cells must pass through a highly organized differentiation process to produce haploid sperm. Spermatogenesis is characterized by three main phases: a mitotic amplification phase, a meiotic phase and a post-meiotic phase also known as spermiogenesis. Within the *Drosophila* testis 50% of the protein coding genes become activated, and among them many in a testis-specific or testis-enriched manner [1], [2]. Since most transcription ceases with entry into the meiotic divisions, spermiogenesis is mainly based on translationally repressed and stored mRNAs (e.g. mRNAs that encode protamines) transcribed in a prolonged meiotic prophase in primary spermatocytes [3]–[5]. Primary spermatocytes are highly transcriptionally active cells that produce two different types of mRNAs: those required for the primary spermatocytes themselves and those that encode proteins necessary for spermiogenesis [2]. Transcription of spermiogenesis-relevant genes depends on the “meiotic arrest” genes that belong to the *aly*-class or the *can*-class [6]. Proteins of the *aly*-class exhibit a broader range of target genes and are part of the testis-specific meiotic arrest complex (tMAC) [7]. The *can*-class comprises proteins specifically expressed in the testis and homologous to the TATA box binding protein-associated factors (TAFs). To date, five testis-specific TAFs (tTAFs) have been described: Cannonball (Can; dTAF5 homolog), No hitter (Nht; dTAF4 homolog), Meiosis I arrest (Mia; dTAF6 homolog), Spermatocyte arrest (Sa; dTAF8 homolog) and Ryan express (Rye; dTAF12 homolog) [8], [9]. In addition, TAF1-2, an isoform of TAF1 is expressed in primary spermatocytes and together with tTAFs and other factors might form a testis-specific TFIID complex [10].

In primary spermatocytes, large amounts of tTAFs and TAF1 localize to the nucleolus [10], [11]. In addition to gene activation, a second function for tTAFs as a repressor of the Polycomb Repression Complex 1 (PRC1) by recruitment of PRC1 to the nucleolus has been proposed [11]. However, recently published genome-wide and cell-specific analyses argue against a model where Polycomb displacement is involved in gene activation in spermatogenesis [12]. In primary spermatocytes, the “meiotic arrest” genes are known to activate directly or indirectly about 1500–2000 spermiogenesis-relevant genes whose mRNAs become translationally repressed until post-meiotic stages. In addition, the “meiotic arrest” genes are essential for cell cycle progression and mutant males show a meiotic arrest phenotype [1]. Based on the fact that these mutants lack post-meiotic stages, the impact of the “meiotic arrest” genes for spermiogenesis is difficult to address. To date, the tMAC and the testis-specific TFIID complex are the only two described transcription complexes in *Drosophila* spermatogenesis. Hence, many open questions remain, e.g. the transcriptional co-factors are not known, it is not known if and how both complexes interact, or whether additional transcription complexes exist in primary spermatocytes. Our aim was to discover which epigenetic “reader” proteins cooperate with tTAFs in primary spermatoyctes.

Acetylation of N-terminal histone tails by histone acetyltransferases (HATs) plays an important role in gene regulation [13]. Acetylation of lysine residues on histone tails is linked to gene activation and can be recognized by bromodomain-containing proteins (BRDs) [14]–[16]. The bromodomain is a module of about 110 amino acids that is conserved in many chromatin-associated proteins including HATs and ATP-dependent chromatin-remodeling factors [17]. Nearly all HAT-associated transcriptional co-factors, such as GCN5, p300/CBP, p300/CBP-associated factors, as well as TAF1 contain bromodomains [18].

Previously, we identified the testis-specifically expressed double bromodomain-containing protein tBRD-1 that is essential for male fertility [19]. In primary spermatocytes, tBRD-1 partially co-localizes with tTAFs and TAF1 in a tTAF dependent manner. Although, tBRD-1 expression is restricted to primary spermatocytes, *tbrd-1* mutants passed through meiosis and only exhibit a post-meiotic phenotype. This is in clear contrast to tTAF mutants that show a meiotic arrest phenotype and completely lack post-meiotic germ cells. A more detailed investigation of *tbrd-1* mutants showed that post-meiotic spermatid nuclei failed to elongate. In addition, the typical clustered arrangement of 64 spermatid nuclei within one cyst was disturbed in *tbrd-1* mutant testes and spermatids failed to individualize. However, spermiogenesis was not completely disturbed since for example the histone-to-protamine transition took place in *tbrd-1* mutants and elongated flagella could be observed. This indicated that tBRD-1 plays an important role in primary spermatocytes that is crucial for specific steps during spermiogenesis. We previously proposed that in primary spermatoyctes, tBRD-1 might act together with tTAFs to activate transcription of a subset of genes that encode proteins with relevance for spermiogenesis [19].

A special group of bromodomain proteins is the bromodomain and extra-terminal (BET) family characterized by having one (plants) or two (animals/yeast) N-terminal bromodomains and a poorly characterized extra-terminal (ET) domain assumed to function as a protein-protein interaction motif. The ET domain comprises three different regions: a conserved NET domain (for N-terminal ET), an intervening sequence and a C-terminal SEED motif. Plant BET proteins often lack the SEED domain.

After bromodomain-mediated association of BET proteins with acetylated chromatin, the ET domain may function as an interface to localize different complexes or proteins to chromatin. BET proteins associate with chromatin and with the basal transcriptional machinery to modulate chromatin structure and influence transcription in a sequence-independent manner [20], [21]. In mammals, BRD2 is involved in gene activation, e.g. by binding to and recruiting the TATA box binding protein (TBP) [22]. The ET domain of BRD4 regulates transcriptional activity by e.g. recruiting specific factors to chromatin [23]. In *Drosophila*, the only known BET gene so far is *female sterile (1) homeotic* (*fs(1)h*) that encodes a multifunctional transcriptional regulator crucial for establishing and maintaining cell fates [24].

To confirm that tBRD-1 is involved in gene expression, we performed microarray analyses and showed that in *tbrd-1* mutant testes hundreds of transcripts are indeed significantly down-regulated compared to levels in wild-type testes. Additionally, we show that a *tbrd-1-eGFP* transgene constructed from the genomic region not only reverses the male sterile phenotype of *tbrd-1* mutants but also the effects on gene activity. Subsequently, we compared the transcriptomes of *tbrd-1* mutant testes with those of *sa* mutants and demonstrate a significant overlap of target genes. Furthermore, we identified two new BET genes in *Drosophila*: *tbrd-2* and *tbrd-3*. Both encoded proteins are testis-specifically expressed and partially co-localize with tTAFs and tBRD-1 in primary spermatocytes. The subcellular localization of tBRD-2 and tBRD-3 is dependent on the acetylation status within the cell. Moreover, similar to plant BET proteins, tBRD-2 and tBRD-3 exhibit only one bromodomain. Thus, we present here for the first time existence of BET family proteins containing a single bromodomain in animals. In addition, we demonstrate that tBRD-1 is able to homodimerize and form heterodimers with tBRD-2, tBRD-3 and tTAFs. These data confirm our hypothesis that tBRD-1 controls gene activity in male germ cells and we propose a model of how tBRDs together with tTAFs might control gene expression in primary spermatocytes.

## Results

### tBRD-1 is required for gene expression in the testis

Previously, we showed that tBRD-1 partially co-localizes with tTAFs in primary spermatocytes. While tBRD-1 expression is restricted to primary spermatocytes, the lack of tBRD-1 function only has consequences in post-meiotic stages. Therefore, we proposed that tBRD-1 acts together with tTAFs to initiate transcription of a special subset of genes with relevance for spermiogenesis [19].

**Figure 1:**
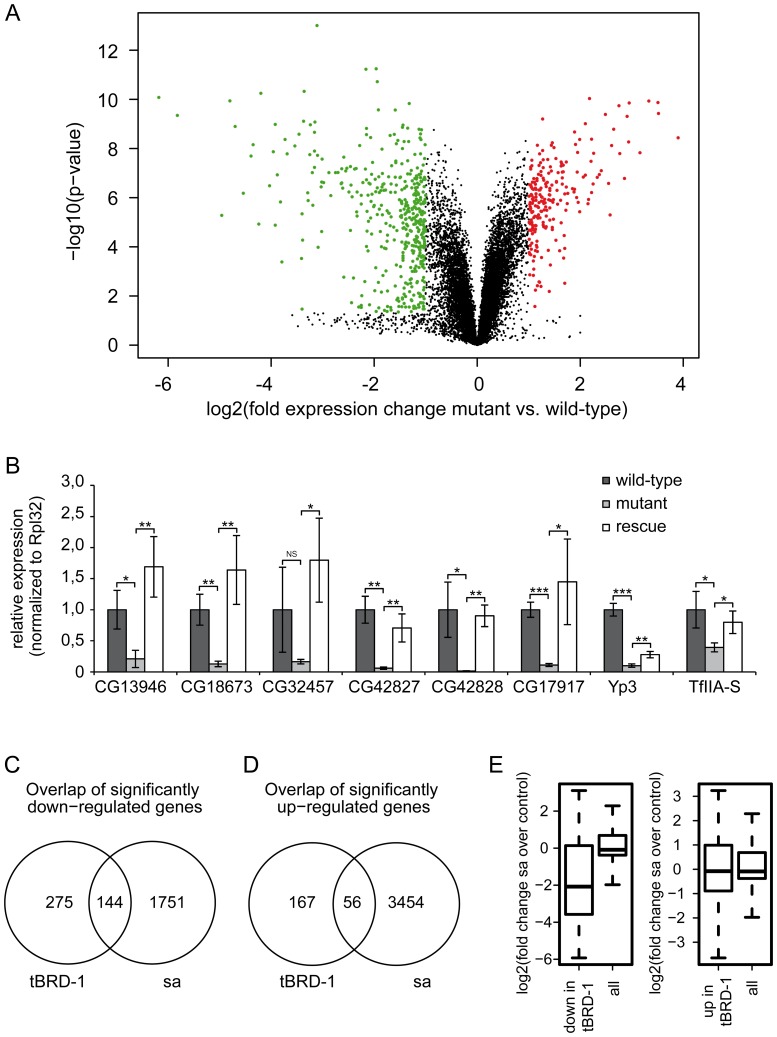
tBRD-1 is required for gene activity in the testis. (A) Distribution of gene expression changes observed when comparing wild-type and *tbrd-1* mutant testis. The volcano plot shows the logarithm of the probability of the t-test as a function of the logarithm of fold change for each reporter on the microarray. Reporter with an absolute log2-fold change ≥1 and a corrected p-value ≤0.05 are plotted in red (up-regulated) and green (down-regulated). (B) Quantitative real-time PCR (qPCR) using cDNA of wild-type, *tbrd-1^1^* and *tbrd-1-eGFP*; *tbrd-1^1^* testes. Transcript levels of *CG13946*, *CG18673*, *CG32457*, *CG42827*, *CG42828, CG17917*, *Yp3* and *TfIIA-S* were significantly reduced in *tbrd-1* mutant testes compared to wild-type and *tbrd-1-eGFP*; *tbrd-1^1^* testes. P-values for significance: * p≤0.05, ** p≤0.01 and *** p≤0.001. NS: not significant. (C,D) Significantly changed genes in *tbrd-1* mutants and *sa* mutants. (C) 144 genes were significantly down-regulated in both *tbrd-1* and *sa* mutants. (D) 56 genes were significantly up-regulated in *tbrd-1* and *sa* mutants. (E) Genes down-regulated (left boxplot) and up-regulated (right boxplot) in *tbrd-1* mutant tested were analyzed for the associated transcriptional changes observed in *sa* mutant testes (all: all genes).

To analyze whether tBRD-1 is indeed involved in gene expression, we performed microarray analyses, and compared the testes transcriptomes of homozygous *tbrd-1* mutants (*tbrd-1^1^*) and wild-type flies (Figure 1). Five independent wild-type and five independent *tbrd-1* mutant hybridizations were carried out using Affymetrix *Drosophila* Genome 2.0 arrays. For each array, independent RNA from whole testes pooled from 25 animals was used. After quality control and normalization the expression values for each probe set from the five arrays of the same genotype were averaged and the log2-fold change between mutant and wild-type was calculated. A positive log2-fold change corresponds to elevated transcription in *tbrd-1* mutants. Genes with a log2-fold change ≥+1 or ≤−1 were considered to have altered expression. Probe set specific P-values were corrected for testing multiple hypotheses (corrPVal) and used in place of P-value to assess the significance of altered gene expression. At a corrPVal of ≤0.05 642 probe sets showed a statistically relevant change in expression. Of those, 223 genes were up-regulated and 419 genes were down-regulated (Figure S1A and Dataset S1). Raw data were deposited in the GEO database (accession number GSE52511).

Obviously, tBRD-1 can function in either way: gene activation and repression. The *tbrd-1^1^* allele was generated by remobilization and subsequent integration of P(EPgy2)^EY02323^ into the *tbrd-1* gene [19]. As expected *tbrd-1* itself was significantly down-regulated in *tbrd-1* mutant testes. In addition, in *tbrd-1* mutant testes a significant up-regulation of the *white* gene was detectable due to the integration of P(EPgy2)^EY02323^ (bearing the *white* gene) in *tbrd-1^1^* (Dataset S1). Quantitative real-time PCR (qPCR) using cDNA of wild-type and *tbrd-1^1^* testes was carried out to validate the results of the microarray experiments (Figure 1B and Figure S1B).

Recently, we showed that *tbrd-1^1^* homozygous males and *tbrd-1^1^/Df(3R)ED10893* or *tbrd-1^1^/Df(3R)Exel9014* trans-heterozygous males shared the same phenotype. In addition, we showed that expression of the fusion protein tBRD-1-eGFP in *tbrd-1^1^* mutants rescues male sterility [19]. Nevertheless, to exclude a “second hit” on the *tbrd-1^1^* mutant chromosome, qPCRs using cDNA from *tbrd-1-eGFP*; *tbrd-1^1^* testes were also performed here. Transcript levels of *CG13946*, *CG18673*, *CG32457*, *CG42827*, *CG42828* (note: annotation *CG6784* split into *CG42827* and *CG42828* in release 5.30 of the genome annotation FlyBase Genome Annotators, 2010), *CG17917*, *Yp3*, and *TfIIA-S* were significantly reduced in *tbrd-1* mutant testes compared to wild-type and *tbrd-1-eGFP*; *tbrd-1^1^* testes (Figure 1B). Clearly, the reduced transcript levels are the result of the *tbrd-1* mutation since testes of *tbrd-1-eGFP*; *tbrd-1^1^* flies exhibit similar or even higher transcript levels than wild-type testes.

*TfIIA-S* is the neighboring gene of *tbrd-1* and encodes a transcription factor. We thus performed qPCRs to exclude that the *TfIIA-S* gene region itself is affected (by remobilization and subsequent integration of P(EPgy2)^EY02323^) in *tbrd-1* mutants. However, the transcript levels of *TfIIA-S* were reduced in *tbrd-1^1^* testes but not in *tbrd-1-eGFP*; *tbrd-1^1^* testes (Figure 1B). Apparently remobilization and subsequent integration of P(EPgy2)^EY02323^ in *tbrd-1^1^* flies did not affect the *TfIIA-S* gene itself. In addition, qPCRs revealed that the transcript levels of up-regulated genes such as *CG31750* (note: formerly known as *Gr36d*; FlyBase curator comment: Gene symbol reverted back to CG symbol because this gene is no longer considered a member of the gustatory receptor (Gr) superfamily [25]), *cutlet*, *TwdlV* and *CG1441* were enriched in *tbrd-1* mutant testes compared to wild-type and *tbrd-1-eGFP*; *tbrd-1^1^* testes (Figure S1B).

Our microarray analyses showed that tBRD-1 is directly or indirectly involved in gene expression within the testis. Here, we concentrated on the down-regulated probe sets that reflect genes directly or indirectly activated by tBRD-1. 391 of the 419 down-regulated probe sets matched annotated protein-coding genes in FlyBase [26]. Among these 391 probe sets 157 (40.2%) corresponded to a characterized gene having a specific name, while 234 (59.8%) matched only to CG numbers. To determine when the corresponding transcripts are detectable during spermatogenesis the 391 probe sets were analyzed using the “*Drosophila spermatogenesis expression database*” (http://mnlab.uchicago.edu/sppress/) [27]. This analysis revealed that 66.2% of the transcripts were enriched in post-meiotic male germ cells and 21.2% in meiotic cells. A few transcripts (8.7%) were enriched in mitotic cells and 3.8% could not be found in this database.

Our microarray data nicely demonstrated that tBRD-1 is indeed involved in gene activation in the testis and the majority of the corresponding transcripts accumulated in post-meiotic cells. Since tBRD-1 expression is restricted to primary spermatocytes we propose that in these cells tBRD-1 is mainly required to activate genes whose transcripts become translationally repressed until post-meiotic stages. This fits well with the post-meiotic phenotype of *tbrd-1* mutants.

Next, we compared our microarray data with available data sets of *sa* mutant testes (GSE48837) [28]. We observed big differences between both transcriptomes, but also a subset of common target genes (Figure 1C,D). In *tbrd-1* mutant testes, 419 genes were significantly down-regulated. Of these 144 (34.4%) were also down-regulated in *sa* mutant testis (Figure 1C). In contrast, among the 223 significantly up-regulated *tbrd-1* target genes only 56 genes (24.1%) were also up-regulated in *sa* mutant testes (Figure 1D). We determined the significance of the observed overlaps using the hypergeometric probability distribution. In case of the up-regulated genes we only found a modest albeit significant enrichment of the observed overlap over the expected overlap (1.3-fold, p<1.22e-05). In contrast, we observed a 3.4-fold enrichment in case of the down-regulated genes (p<3.55e-41). Similarly, we analyzed the relationship between genes down-regulated in *tbrd-1* and *sa* mutant testes in a more quantitative fashion (Figure 1E). In general, genes down-regulated in *tbrd-1* mutant testes were also down-regulated in *sa* mutant testes (Figure 1E, left). In contrast, genes up-regulated in *tbrd-1* mutant testes were not specifically de-regulated in *sa* mutant testes (Figure 1E, right). Indeed, tBRD-1 and Sa share a subset of common target genes, implying that tBRD-1 acts together with tTAFs to activate a subset of genes with relevance for spermiogenesis.

### tBRD-2 and tBRD-3 constitute two new types of BET proteins in *Drosophila*

**Figure 2:**
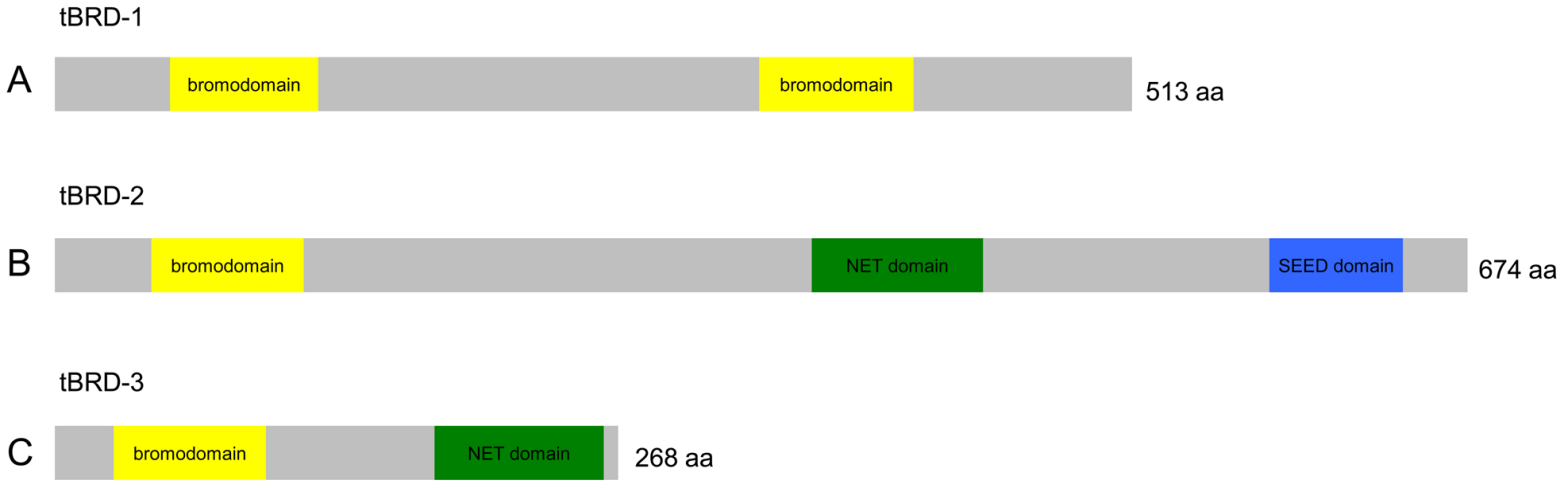
tBRD-2 and tBRD-3 represent two new types of BET proteins. Scheme of full-length tBRD-1 (A), tBRD-2 (B) and tBRD-3 (C) proteins. Bromodomains are indicated in yellow, NET domains in green and the SEED domain in blue.

Besides tBRD-1 with two widely spaced bromodomains (amino acid 55 to 127 and 336 to 409) (Figure 2A and [19]) the *Drosophila* genome encodes two further as yet uncharacterized testis-specific bromodomain containing proteins. The *Drosophila CG7229* gene encodes a 674 amino acid protein of 74.6 kDa (Flybase) [26] with a predicted N-terminal bromodomain (amino acid 47 to 119), a NET domain (amino acid 362 to 443) (Prosite database) [29] and a SEED motif (amino acid 580 to 660) [20] (Figure 2B). The *Drosophila CG30417* gene encodes a 268 amino acid protein of 30.6 kDa (Flybase) [26] with a predicted N-terminal bromodomain (amino acid 28 to 100) and a NET domain (amino acid 181 to 261) (Prosite database) [29] (Figure 2C). Beyond the N-terminal bromodomains and the NET domains all three tBRD proteins show no sequence similarity.

RT-PCR analyses revealed that *CG7229* and *CG30417* transcripts are predominantly detectable in male gonads (Figure S2A,B). Affymetrix expression data also indicate an enrichment of both transcripts in the testis (Flyatlas: the *Drosophila* gene expression atlas) [30]. Consequently, we renamed CG7229 tBRD-2 and CG30417 tBRD-3 (testis-specifically expressed bromodomain-containing protein-2 and 3) and the corresponding genes *tbrd-2* and *tbrd-3*. In animals, proteins of the BET family have two bromodomains and an ET domain [20]. tBRD-2 and tBRD-3 are different since both have only a single N-terminal bromodomain. Additionally, tBRD-2 has a typical ET domain comprising a NET domain, an intervening sequence and a C-terminal SEED motif, whereas tBRD-3 has only the conserved NET domain of the ET domain. Beyond the bromodomains and the ET domains, tBRD-2 and tBRD-3 proteins show no sequence similarity to human or plant BET protein family members. We propose that tBRD-2 and tBRD-3 constitute two new members of the BET protein family in *Drosophila* that more resemble BET proteins in plants.

### tBRD-1, tBRD-2 and tBRD-3 largely co-localize at the chromosome territories in primary spermatocytes

**Figure 3:**
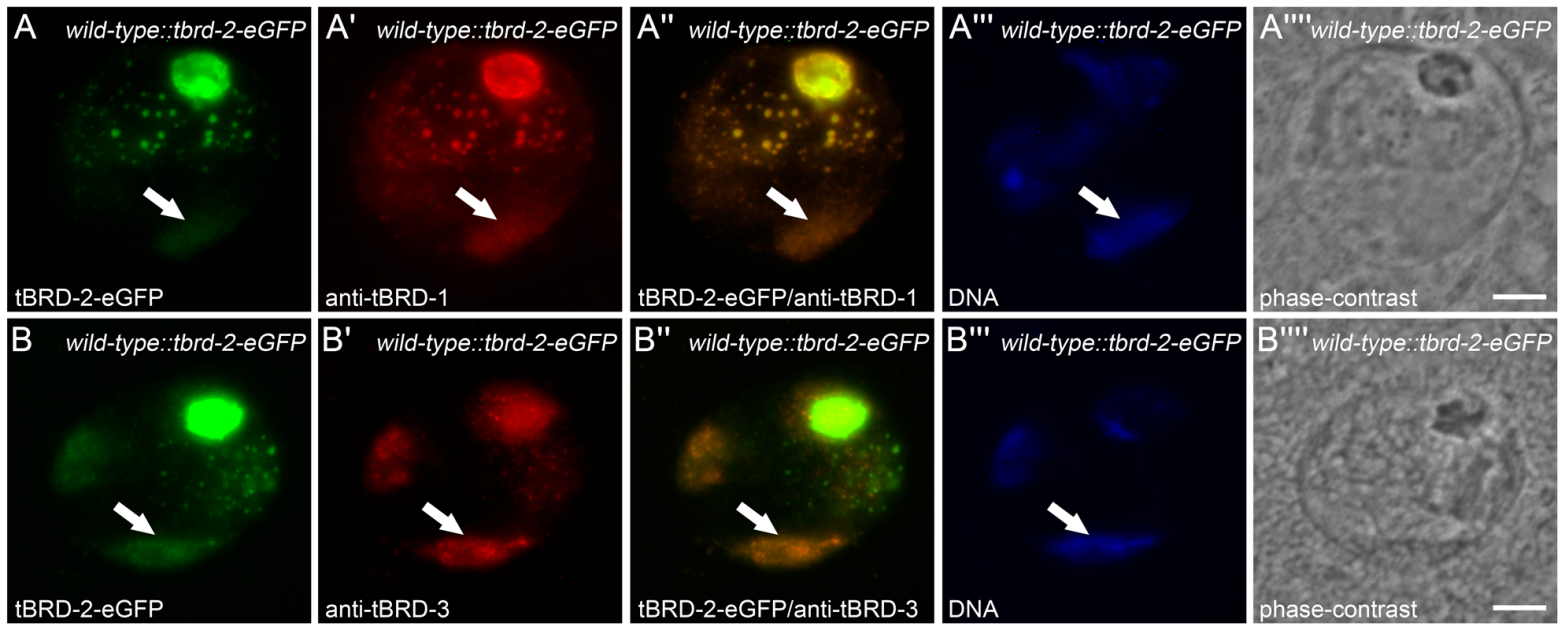
In primary spermatocytes tBRD-2 co-localizes with tBRD-1 and tBRD-3. Single primary spermatocyte nuclei of flies expressing tBRD-2-eGFP stained with anti-tBRD-1 (A panels) or anti-tBRD-3 (B panels). (A,B) tBRD-2-eGFP was visible over the chromosome territories (arrows). tBRD-2-eGFP partially co-localizes with tBRD-1 (A″) and tBRD-3 (B″) over the chromosomes (arrows). (A′″,B′″) Hoechst DNA staining. (A″″,B″″) Phase-contrast images. Scale bars: 5 µm.

Recently, we showed that tBRD-1 is specifically expressed in primary spermatocytes, strongly localizing to the nucleolus, with lower amounts distributed over the partially condensed chromosomes [19] (Figure 3A′). Within the nucleoplasm tBRD-1 localizes to several nuclear speckles of unknown function. Within the scope of the experiments described here we focused on the chromosomal localization of tBRD-1, tBRD-2 and tBRD-3 in primary spermatocytes. To analyze the subcellular localization of tBRD-2 we generated *tbrd-2-eGFP* transgenic flies that express the fusion protein under the control of the endogenous gene regulatory regions. To analyze tBRD-3 we raised a peptide antibody (aa 175 to aa 189). The specificity of the antibody was confirmed by a peptide-blocking assay (Figure S3).

**Figure 4:**
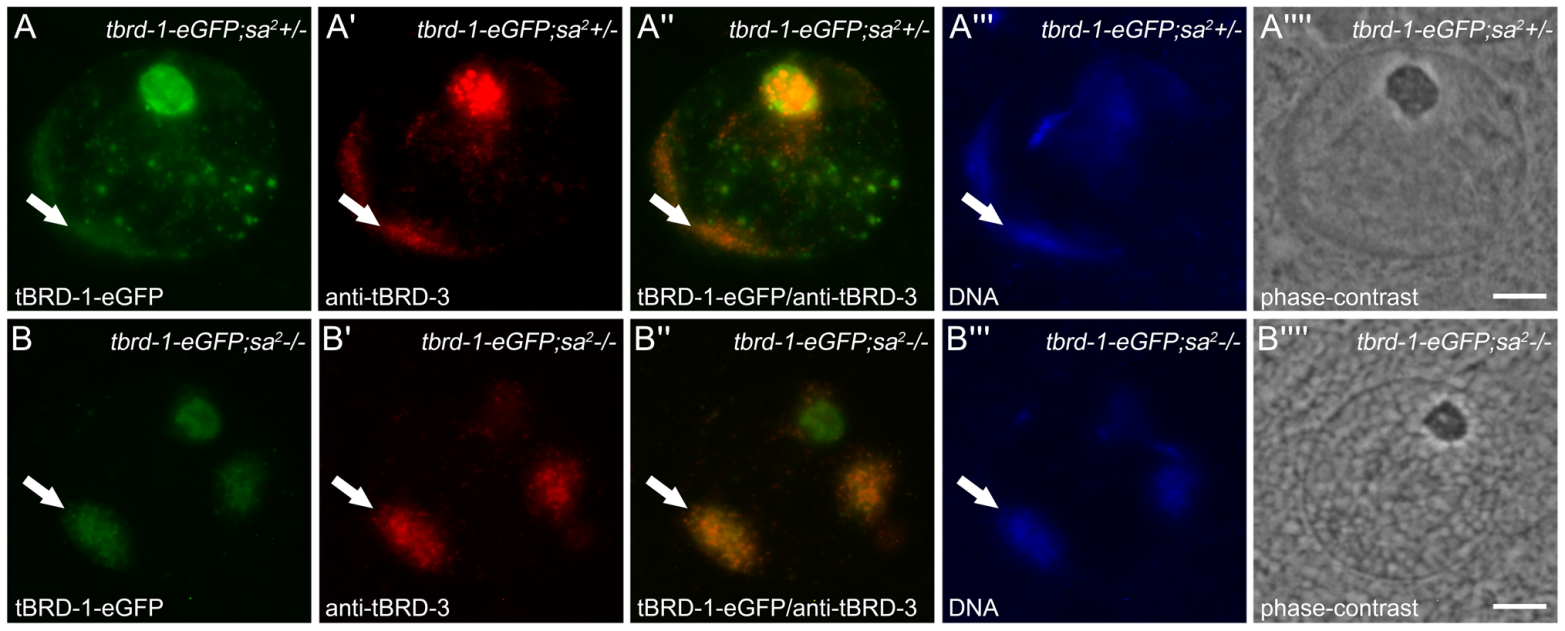
Recruitment of tBRD-1 and tBRD-3 to chromatin is independent of tTAF Sa. Single primary spermatocytes from heterozygous (A panels) and homozygous *sa^2^* (B panels) mutants that express tBRD-1-eGFP stained with anti-tBRD-3 antibody. (A″,B″) In heterozygous and homozygous *sa^2^* mutant spermatocytes tBRD-3 partially co-localizes with tBRD-1-eGFP over the chromosomes (arrows). (A′″,B′″) Hoechst DNA staining. (A″″,B″″) Phase-contrast images. Scale bars: 5 µm.

Examination of mature spermatocytes expressing tBRD-2-eGFP (Figure 3A,B) or immunostained with anti-tBRD-3 (Figure 3B′) revealed that, like tBRD-1, tBRD-2 and tBRD-3 localize to the partially condensed chromosomes (Figure 3A,B,B′, arrows). Thus, in primary spermatocytes tBRD-1, tBRD-2 and tBRD-3 largely co-localize and might cooperate in transcription of spermatogenesis-relevant genes. Recently, we could show that proper protein distribution of tBRD-1 in spermatocytes required wild-type function of tTAFs [19]. Likewise, immunofluorescence staining of *sa^2^* mutant testes showed that localization of tBRD-2-eGFP and tBRD-3 is altered in homozygous *sa^2^* mutant spermatocytes (Figure S4B and Figure 4B′). The localization to the nucleolus was strongly reduced, increased signals were visible within chromosome territories and nuclear speckles were hardly detectable. However, despite the altered distribution of tBRD-1, tBRD-2 and tBRD-3, co-localization of tBRD-2 and tBRD-3 with tBRD-1 was still detectable over the chromosomes in homozygous *sa^2^* mutant testes (Figure S4B″ and Figure 4B″, arrows). Clearly, tTAF Sa is not required for recruiting tBRD-1, tBRD-2 or tBRD-3 to chromosomes.

**Figure 5:**
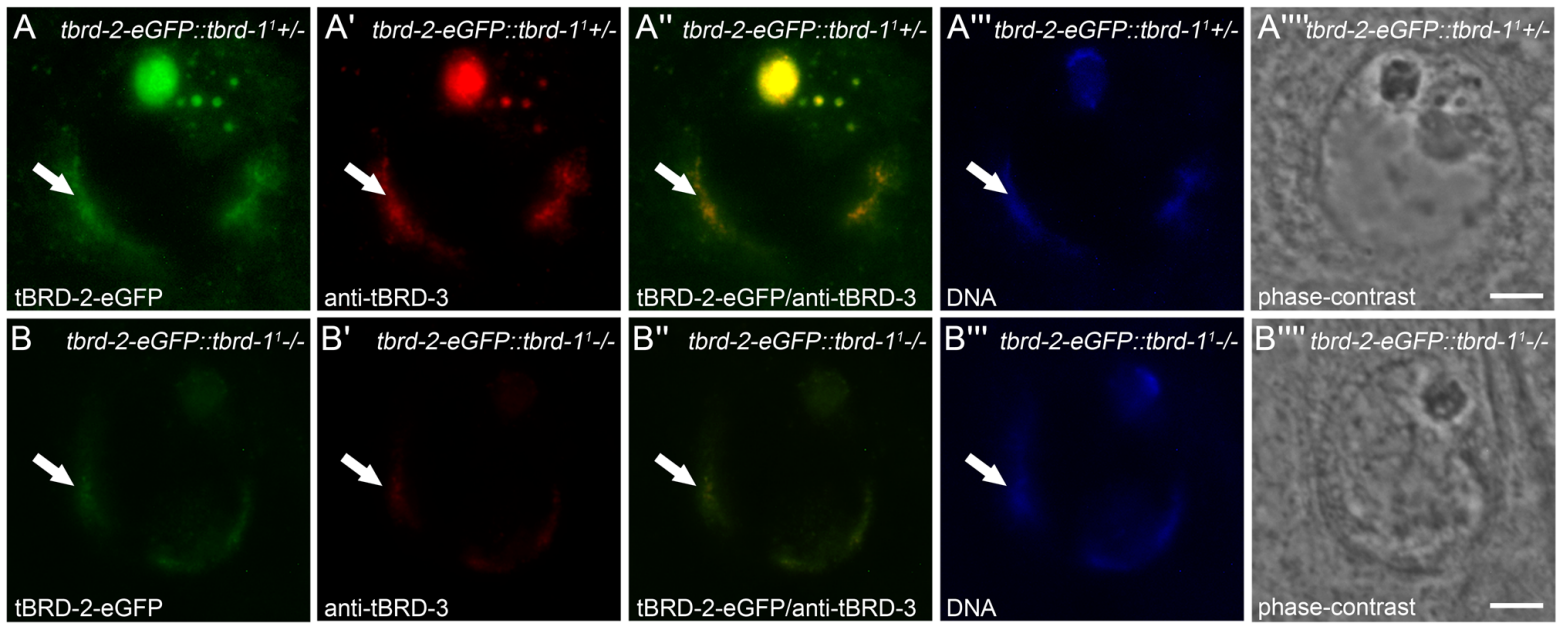
tBRD-1 is not essential for co-localization of tBRD-2 and tBRD-3. Single primary spermatocytes from heterozygous (A panels) and homozygous *tbrd-1^1^* (B panels) mutants that express tBRD-2-eGFP stained with anti-tBRD-3 antibody. (B″) Partial co-localization of tBRD-2-eGFP and tBRD-3 over the chromosomes (arrows) was still detectable in homozygous *tbrd-1^1^* mutant spermatocytes. (A′″,B′″) Hoechst DNA staining. (A″″,B″″) Phase-contrast images. Scale bars: 5 µm.

Analyses of homozygous *tbrd-1* mutant testes demonstrated that protein distributions of tBRD-2 and tBRD-3 are altered in primary spermatocytes (Figure 5B,B′). As above, localization to the nucleolus was strongly reduced and nuclear speckles were hardly detectable. However, both proteins continued to co-localize over the chromosomes, although in a severely reduced manner (Figure 5B–B″, arrows) (note: *tbrd-2* and *tbrd-3* transcripts are not significantly down-regulated in *tbrd-1* mutant testes). Obviously, tBRD-1 is not absolutely essential for recruitment of tBRD-2 and tBRD-3 to chromosomes and their co-localization, but might increase and/or stabilize their binding to chromatin.

### The acetylation status influences the localization of tBRD-1, tBRD-2 and tBRD-3 in primary spermatocytes

**Figure 6:**
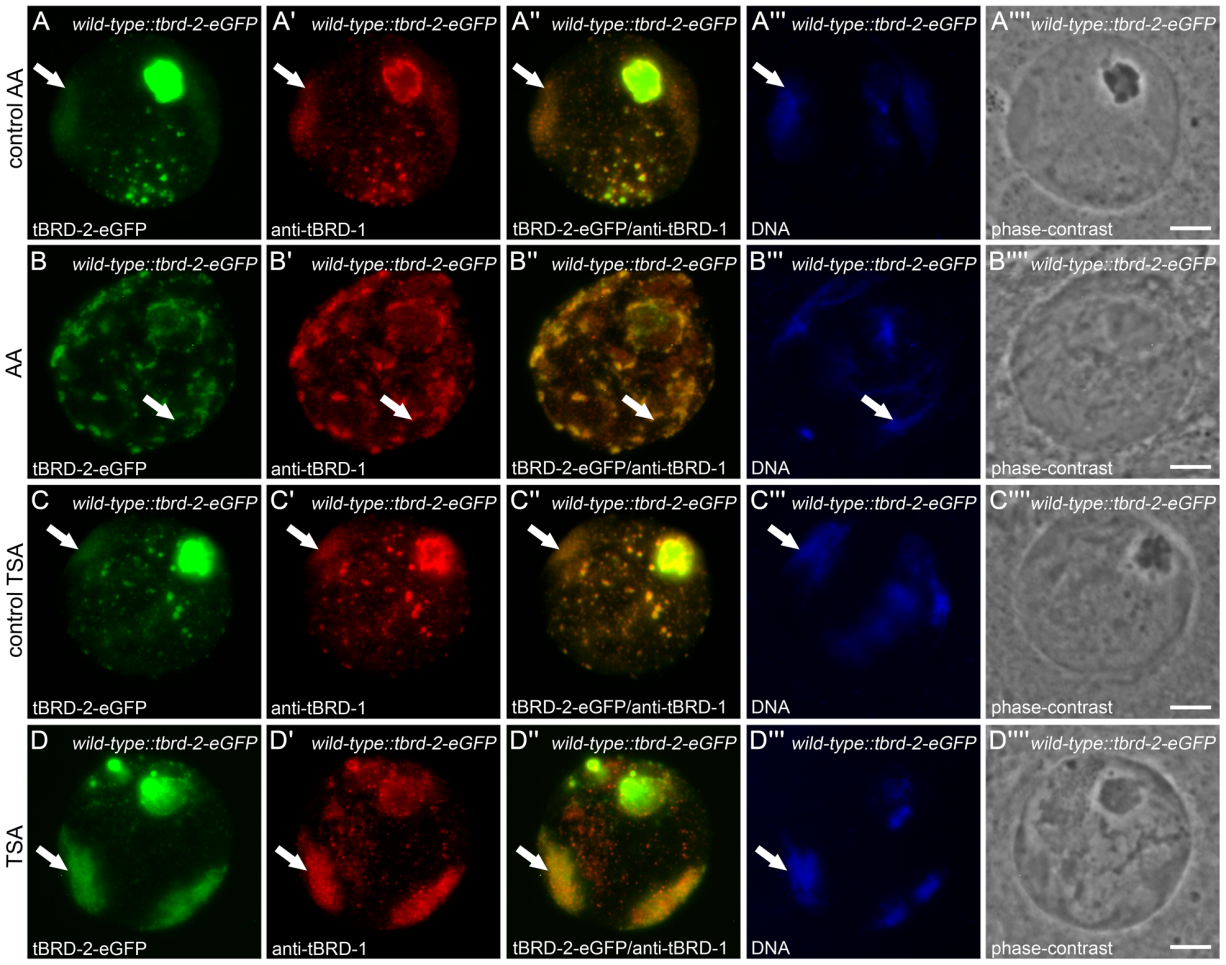
Acetylation levels in primary spermatocytes influences tBRD-2-eGFP localization. Pupal testis of tBRD-2-eGFP expressing flies were treated with anacardic acid (AA) (B panels) or TSA (D panels) for 24 hours in culture and afterwards spermatoyctes were stained with an antibody against tBRD-1. (A and C panels) Untreated control. (B) Incubation of testis with AA led to a spotted pattern of tBRD-2-eGFP at chromosome territories (arrow) compared to the control (A). (D) TSA treatment led to increased localization of tBRD-2-eGFP to the chromosomes (arrow) compared to the control (C). (B″,D″) Partial co-localization of tBRD-2-eGFP and tBRD-1 was not affected by AA or TSA treatment. (A′″,B′″,C′″,D′″) Hoechst DNA staining. (A″″,B″″,C″″,D″″) Phase-contrast images. Scale bars: 5 µm.

Previously, we could show that the distribution of tBRD-1 in primary spermatocytes changed after inhibitor treatment of cultured germ cells with trichostatin A (TSA) or anachardic acid (AA) [19]. Treatment of cells with TSA inhibits histone deacetylases (HDACs) and leads to a significant increase of histone H4 acetylation in primary spermatocytes, while AA leads to a dramatic decrease in acetylation by inhibiting histone acetyltransferases (HATs) [19], [31], [32]. Here, we treated pupal testes of *tbrd-2-eGFP* transgenic flies with either TSA or AA and subsequently performed immunofluorescence staining using an anti-tBRD-1 antibody (Figure 6). Increased or decreased acetylation was confirmed by an anti-histone H4ac antibody (Figure S5).

After treatment with AA, tBRD-2-eGFP and tBRD-1 distribution at the chromosomes was dramatically altered (Figure 6B,B′, arrows) compared to control spermatocytes (Figure 6A,A′, arrows). Treatment with TSA led to increased tBRD-2-eGFP and tBRD-1 localization to the chromosomes (Figure 6D,D′, arrows). Interestingly, an increase or decrease in acetylation had no effect on the overall co-localization of tBRD-1 and tBRD-2-eGFP (Figure 6B″,D″). This indicates that tBRD-1 and tBRD-2 interact in primary spermatocytes independently from the acetylation level.

Immunofluorescence staining using an anti-tBRD-3 antibody after TSA treatment of pupal testes from *tbrd-1-eGFP* transgenic flies led to similar results (Figure S6); tBRD-3 localization to the chromosomes increased (Figure S6D′, arrow) and co-localization with tBRD-1-eGFP was still visible (Figure S6D″). However, after AA treatment co-localization between tBRD-3 and tBRD-1 was no longer detectable (Figure S6B″). This implies that co-localization of tBRD-1 and tBRD-3 is acetylation dependent. These data indicate that proper recruitment of tBRD-1, tBRD-2 and tBRD-3 to chromatin depends on defined acetylation levels in primary spermatocytes.

### tBRD-1 can interact with tBRD-2, tBRD-3 and tTAFs

**Figure 7:**
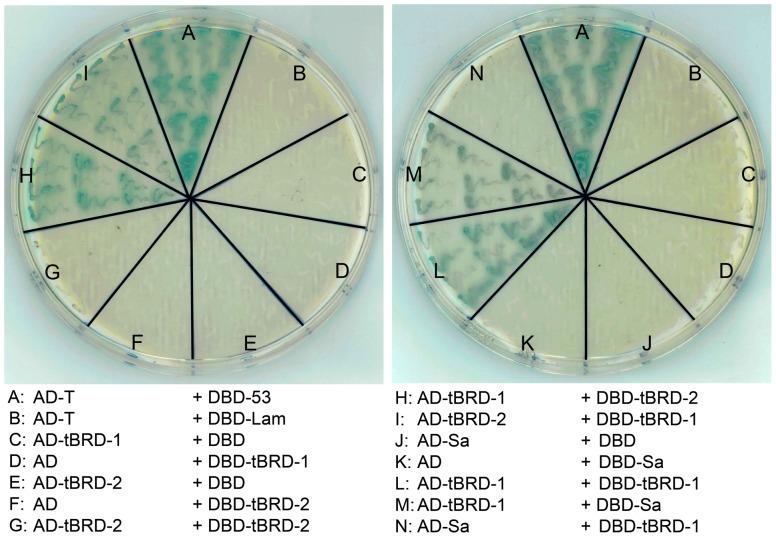
tBRD-1 interacts with tBRD-2 and the tTAF Sa in yeast two-hybrid experiments. (A) Positive control (DBD-53+AD-T). (B) Negative control (DBD-Lam+AD-T). (C–F, J,K) tBRD-1, tBRD-2 and Sa fusion proteins showed no self-activity. (G) No homodimerization of tBRD-2 was detectable. (H,I) tBRD-1 and tBRD-2 heterodimer formation was visible. (L) tBRD-1 was able to homodimerize. (M,N) tBRD-1 and Sa could only interact when Sa was acting as the bait.

The data above point to an interdependency of several tBRDs and tTAFs. Hence, we reasoned that they might interact with each other at the protein level. To analyze whether tBRD-1, tBRD-2, tBRD-3 and the five tTAFs can form homodimers or heterodimers among each other, we performed yeast two-hybrid analyses. Every coding region was inserted into both the pGBKT7 expression vector (expresses a fusion protein of the GAL4 DNA-binding domain (DBD) and a bait protein) and the pGADT7 expression vector (expresses a fusion protein of the GAL4 activation domain (AD) and a prey protein). This enabled us to test each protein acting either as bait or prey. When bait and the prey fusion proteins interact, the DBD and AD are brought into close proximity and subsequently activate reporter genes, e.g. *lacZ*, leading to colony growth and blue color. Some proteins also possess intrinsic DNA-binding and/or transcriptional activating properties (self-activity). Therefore, each DBD-bait and AD-prey fusion protein was also tested in combination with an empty pGADT7 or pGBKT7 expression vector (Figure 7C–F,J,K and Figure S7).

Additionally, a positive (DBD-53+AD-T) and a negative (DBD-Lam+AD-T) control was included on each plate (Figure 7 and Figure S7). DBD-53 encodes a fusion protein between the DBD and murine p53, AD-T encodes a fusion protein between the AD and the SV40 large T-antigen, and DBD-Lam encodes a fusion protein between the DBD and human lamin C. SV40 large T-antigen is known to interact in yeast two-hybrid assays with murine p53 [33], [34] but not with Lamin C [35], [36]. With the exception of DBD-Can (Figure S7E) no self-activity was observed (Figure 7C–F,J,K and Table S1 and Figure S7).

tBRD-1 was able to form homodimers (Figure 7L and Table S2) as well as heterodimers with tBRD-2 (Figure 7H,I and Table S2), tBRD-3 (Figure S7A and Table S2), Sa (Figure 7M and Table S3), Rye (Figure S7D and Table S3) and Can (Figure S7E,G and Table S3). Although we observed self-activity potential for DBD-Can the growth of blue colonies was much lower for DBD-Can in combination with AD than with AD-tBRD-1 (Figure S7E). In addition, heterodimer formation was detectable between tBRD-2 and tBRD-3 as well as between tBRD-3 and Sa (Figure S7B,F and Table S2, S3) and both could interact with the tTAF Rye (Figure S7H and Table S3).

Among the tTAFs homodimerization was only demonstrated for Rye (Figure S7K,L and Table S4). An interaction between Rye and Nht has already been reported [9] and served here as an additional control (Figure S7N and Table S4). An interaction between other tTAFs was not observed (Figure S7 and Table S4). We never observed interactions with Mia fusion proteins. However, some fusion proteins are not stably expressed in yeast. Therefore, we do not know if Mia does not interact with tBRDs and/or other tTAFs or if Mia fusion proteins are not stably expressed.

**Figure 8:**
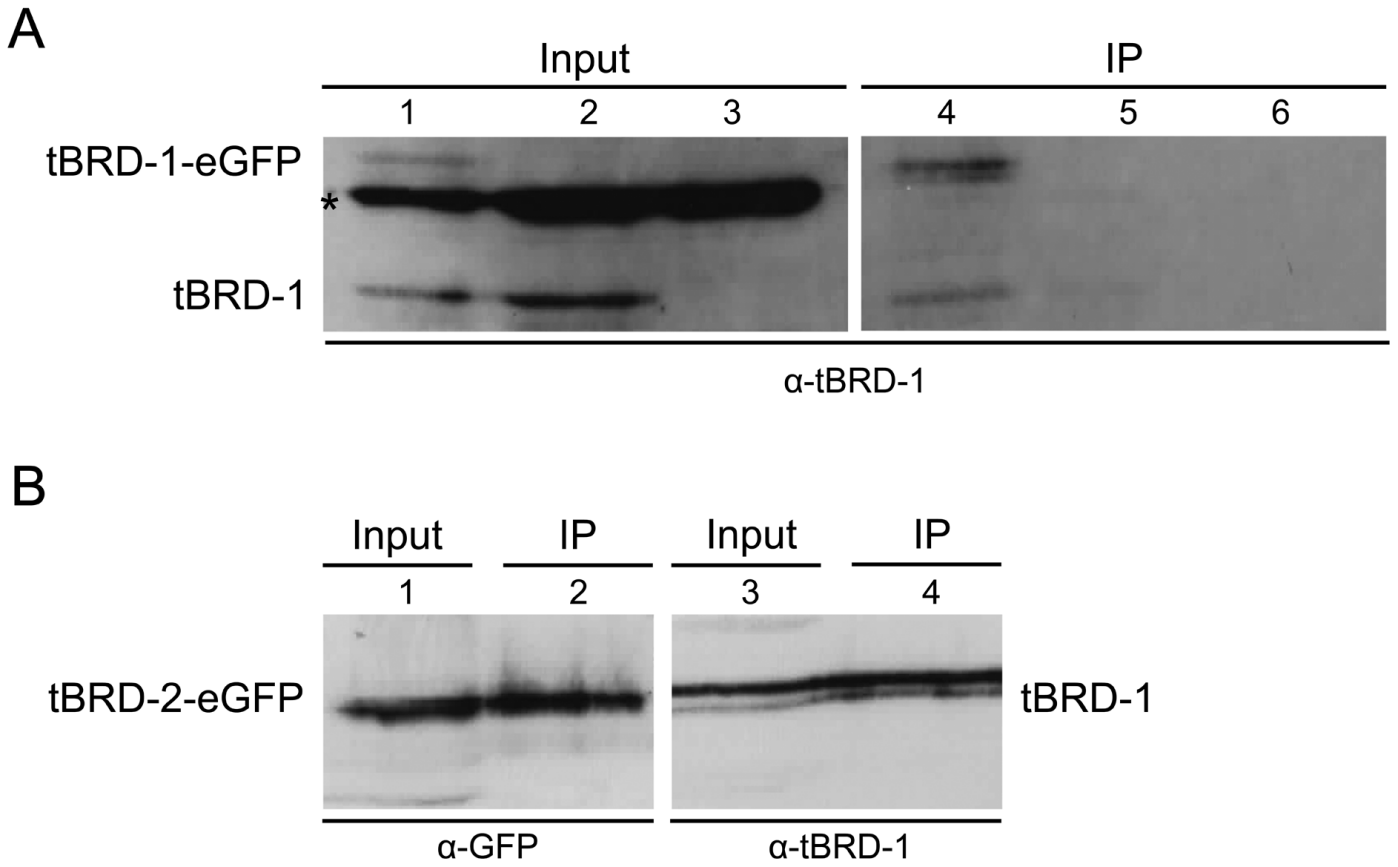
tBRD-1 co-precipitates with tBRD-1-eGFP and tBRD-2-eGFP from testes protein extracts. (A) Proteins were analyzed by SDS-PAGE and immunoblotting using anti-tBRD-1 antibody. Lanes 1–3: testes extracts before immunoprecipitation (Input). The anti-tBRD-1 antibody detected a protein at about 56 kDa (tBRD-1 predicted molecular mass: 59.2 kDa) in protein extracts of *tbrd-1-eGFP* (lane 1) and wild-type (lane 2), but not in *tbrd-1* mutant testis (lane 3). Additionally, a protein at about 90 kDa was detected in protein extracts of *tbrd-1-eGFP* testes (lane 1) that represents the tBRD-1-eGFP fusion protein. An unspecific protein at about 76 kDa was visible in all three extracts (asterisk). Lanes 4–6: eGFP-tagged tBRD-1 was immunoprecipitated (IP) with the GFP-Trap A Kit from testes protein extracts of *tbrd-1-eGFP* transgenic flies (lane 4), wild-type flies (lane 5) or *tbrd-1* mutants (lane 6). tBRD-1 was detected in the IP from *tbrd-1-eGFP* testes (lane 4) but not from wild-type (lane 5) or *tbrd-1* mutant testis. (B) Input and immunoprecipitates (IP performed as in A) from testes protein extracts of *tbrd-2-eGFP* transgenic flies were analyzed by SDS-PAGE and immunoblotting using anti-GFP and anti-tBRD-1 antibodies. tBRD-2-eGFP and tBRD-1 were detected in both immunoprecipitates (lanes 2 and 4).

**Figure 9:**
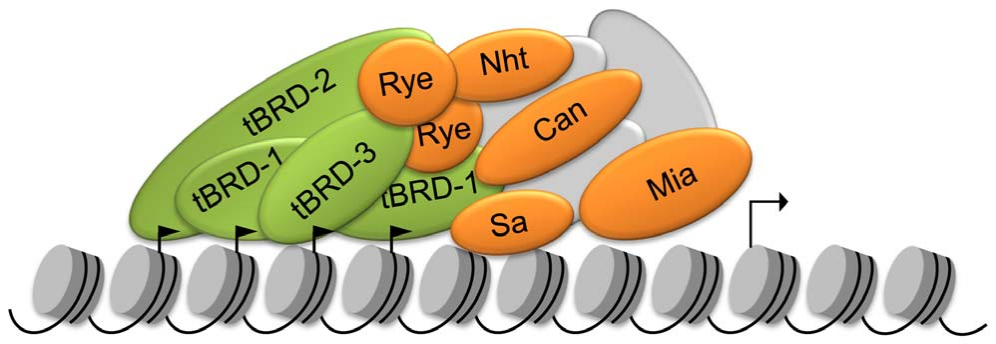
Model for tBRD-1, tBRD-2, tBRD-3 and tTAF function in primary spermatocytes. Scheme of a complex of tBRDs (green), tTAFs (orange) and so far unknown proteins (grey) that may activate transcription of a specific set of genes in primary spermatocytes. In our model tBRDs recognize and bind to acetylated histones (little black flags). This binding could in turn lead to the recruitment of tTAFs and additional transcription factors and subsequently gene activation.

To examine whether homo- and heterodimerization occur within the testis, we performed co-immunoprecipitations (Co-IPs) with testes protein extracts from *tbrd-1-eGFP* and *tbrd-2-eGFP* transgenic flies using the GFP-Trap A Kit. Subsequent immunoblotting revealed that tBRD-1 co-immunoprecipitated with tBRD-1-eGFP and tBRD-2-eGFP (Figure 8). Based on our co-localization studies, inhibitor experiments and protein interaction analyses we hypothesized that a complex comprising tBRD-1, tBRD-2, tBRD-3 and tTAFs exists that controls gene activity in primary spermatocytes (see Figure 9).

## Discussion

Within the testis more than 50% of the *Drosophila* protein coding genes become activated and most of them are transcribed in primary spermatocytes. However, as yet we are far from understanding how gene activation, in particular synthesis of translationally repressed mRNAs, is achieved in male germ cells. Another question is how tTAFs might be connected to chromatin modifications, in particular to acetylated histones recognized by bromodomains. Here, we address the questions of whether tBRD-1 is the only relevant bromodomain protein, whether bromodomain proteins physically interact with tTAFs, whether the recruitment of bromodomain proteins depends on tTAFs and the acetylation status of histones, how many genes depend on the function of tBRD-1 and finally whether common tBRD-1 and tTAF target genes exist.

### tBRD-1, tBRD-2 and tBRD-3 localize to chromatin in primary spermatocytes

Here, we identified two new bromodomain-containing proteins in *Drosophila*. In contrast to tBRD-1, tBRD-2 and tBRD-3 each contain only one bromodomain. However, both exhibit a NET domain a characteristic feature of BET protein family members. We described here for the first time BET proteins in animals that contain only one bromodomain. In primary spermatocytes, both tBRD-2 and tBRD-3 were predominantly expressed in the testis and largely co-localized with tBRD-1 over the chromosomal regions in an acetylation dependent manner. Therefore, we hypothesize that in primary spermatocytes, tBRD-2 and tBRD-3 can act together with tBRD-1 and tTAFs to activate transcription of a subset of spermatogenesis-relevant genes (Figure 9).

### tBRD-1 interact with tBRD-2, tBRD-3 and with tTAFs

Our protein-protein interaction studies indicated that the tTAF Sa, which binds to DNA [11], interacts with tBRD-1. Additionally, we found that tBRD-1 interacts with itself, the tTAFs Rye and Can as well as with tBRD-2 and tBRD-3. We observed that the tTAF Rye can exist as homodimer and is able to form heterodimers with Nht (the Rye and Nht interaction was already shown in [9]) and all three bromodomain proteins. Additionally, interaction of the tTAF Sa with tBRDs was indicated by immunohistology. The overall distribution of tBRDs was altered in *sa* mutant testes. However, despite the altered distribution, co-localization of tBRDs was still detectable. Hence, we propose that Sa function is required for proper subcellular localization of tBRDs, but not essential for their co-localization and principal recruitment of tBRDs to chromatin.

We propose the existence of a transcriptional activating complex that includes all three tBRDs and the TAFs Sa, Can, Rye, Nht and Mia. Additionally, we suggest that the tBRDs, by binding to acetylated chromatin, might act as a scaffold to bundle these proteins for transcriptional activation (Figure 9). However, in the case of Mia we could not observe an interaction with tBRDs or other tTAFs, possibly due to lack of stable Mia fusion proteins in yeast.

### tBRD-1 regulates hundreds of genes

Our microarray analyses showed that tBRD-1 is indeed directly or indirectly involved in gene activation within the testis. In contrast to tTAF mutants, *tbrd-1* mutant testes clearly have post-meiotic cells with elongated flagella [19]. Correspondingly, 66.2% of the tBRD-1 dependent transcripts are enriched in spermatids. Since tBRD-1 expression is restricted to spermatocytes these transcripts likely reflect translationally repressed mRNAs that accumulate in post-meiotic stages, although they are synthesized in primary spermatocytes. This strengthens our hypothesis that in primary spermatocytes, tBRD-1 regulates a subset of the putative 1500–2000 tTAF target genes.

In tTAF mutant testes, genes are affected on a massive scale and several reasons argue against a direct comparison between *tbrd-1* and tTAF mutant testes. tTAF mutant testes are smaller in size and show a meiotic arrest phenotype. Hence, tTAF mutant testes accumulate primary spermatocytes and lack post-meiotic cells [37], [12]. In contrast, *tbrd-1* mutant testes clearly have post-meiotic cells with elongated flagella [19]. The transcriptomes of *tbrd-1* mutant and tTAF mutant testes are likely not directly comparable. Nevertheless, we showed that tBRD-1 and Sa share a common subset of target genes. Among these genes, 144 were significantly down-regulated in *tbrd-1* and *sa* mutant testes. These genes represent a promising starting point for future studies on gene activation in the testis.

### Bromodomain proteins and TAF variants in mammalian spermatogenesis

Developmental differentiation processes based on stored mRNAs or mRNAs synthesized by tissue-specific isoforms of general transcription factors is not a unique feature of *Drosophila* spermatogenesis. Also in mammals, spermiogenesis is programmed by repressed and stored mRNAs. TAF4b, a paralog of TAF4, is highly expressed in mouse testes as well as in ovaries, and is required for male and female fertility [38], [39], [40]. Initially, TAF4b was discovered in a human B-cell line as the first cell-type-specific TFIID subunit [41]. The testis-linked paralog of TAF7, TAF7L, can interact with TBP and other TAFs, and is thought to be part of an alternative TFIID-like complex [42].

Recently, it has been suggested that TAF7L directly cooperates with TBP-related factor 2 (TRF2) to activate a subset of spermiogenesis-relevant genes [43]. The bromodomain protein BRDT (bromodomain testis-specific) is highly enriched in mice testes and essential for male germ cell differentiation and fertility [44], [45]. BRDT specifically binds hyperacetylated histone H4 and exhibits acetylation-dependent chromatin remodeling activity [46]. BRDT can interact with Smarce1, a member of the SWI/SNF family and hyperacetylation of histones increases this interaction [47]. In mice testes, the bromodomain protein BRDT is involved in transcription of a large number of genes [48], [49]. Additionally, the other members of the BET family, BRD2, BRD3 and BRD4, are expressed in mammalian male germ cells [50]. However, it is yet not known whether these BRD proteins interact among each other or with tissue-specifically expressed TAFs.

Our data provide important information and new insights into gene activation in male germ cells that may be relevant to other organisms. We believe that a complex of tBRDs, tTAFs and so far unknown proteins exists that activates transcription of a specific set of genes in primary spermatocytes (Figure 9). In our model tBRDs recognize and bind to acetylated histones, leading to recruitment of tTAFs and additional transcription factors to mediate gene activation.

Many tBRD-1 and Sa target genes overlapped; however, we observed many tBRD-1 targets that do not depend on Sa function and *vice versa*. Therefore, complexes containing either tBRD-1, or tBRD-2 or tBRD-3 in combination with different tTAFs are conceivable. Likewise, complexes containing tBRD-1 and tBRD-2 or tBRD-1 and tBRD-3 or tBRD-2 and tBRD-3 in combination with different tTAFs are possible. These different complexes could further subdivide the many thousands of genes and thus each might activate a certain set of spermatogenesis-relevant genes.

## Materials and Methods

### Fly strains and culture

*Drosophila melanogaster* strains were maintained on standard medium at 25°C. *w^1118^* was used as the wild-type strain. The *tbrd-1* mutant allele, and tBRD-1-eGFP expressing flies were described previously [19]. *sa^2^* mutants [8] were kindly provided by M.T. Fuller (Palo Alto).

### RT-PCR

Total RNA was prepared using TRIzol (Invitrogen) from 80 testes, 40 carcass of males, 40 adult females, and 40 third instar larvae. The OneStep RT-PCR Kit (Qiagen) was used to amplify a 319 bp cDNA fragment from the open reading frame of *tbrd-2*. The chosen primers (*tbrd-2-fw GTCGAACGGCAAAAGTGTTC* and *tbrd-2-rv GGCTCCAAGAAGTCCAAGG*) flank an intron of 54 bp to distinguish between RNA and DNA. To amplify a 462 bp cDNA fragment from the intronless *tbrd-3* polyA^+^-mRNA was prepared from total RNA using Oligotex mRNA Mini Kit (Qiagen). For RT-PCR the following primers were used: *tbrd-3-fw TGGGTTTTCTACGAGCCACT* and *tbrd-3-rv CTGCTCCGAAGTTTCCAAAG*. Primers for the ubiquitously transcribed *β3-tubulin* gene (*b3-tubulin-fw ATCATTTCCGAGGAGCACGGC* and *b3-tubulin-rv GCCCAGCGAGTGCGTCAATTG*) were used as controls. These primers amplify a 372 bp fragment on RNA and a 490 bp fragment on DNA.

### Cloning the *tbrd-2-eGFP* construct

To generate a *tbrd-2-eGFP* construct, the open reading frame (ORF) of the *tbrd-2* gene together with a 591 bp sequence upstream of the ATG translational start was PCR amplified using genomic DNA and primers with linked *EcoRI* and *SpeI* restriction sites. The PCR fragment was inserted into *pChabΔsalΔlacZ-eGFP* in frame with the *eGFP*. Transgenic fly strains were established in a wild-type background.

### RNA purification and microarray analysis

Total RNA was extracted from wild-type testes and homozygous *tbrd-1* mutant testis using TRIzol (Invitrogen). RNA quality was monitored using the Agilent Bioanalyser 2100 with the RNA 6000 Nano kit. Gene expression analysis was carried out using Affymetrix *Drosophila* Genome 2.0 arrays according to the manufacturer's recommendations. For each array, independent RNA from whole testes pooled from 25 animals was used.

### Data deposition

The microarray data from this publication was deposited at NCBI's gene expression omnibus (GEO) under the accession number GSE52511.

### Quantitative real-time PCR

Total RNA from 100 wild-type testes, homozygous *tbrd-1* mutant testes and *tbrd-1* mutant testes that express a tBRD-1-eGFP rescue protein was isolated using TRIzol (Invitrogen). RNA was treated with DNase (Promega) according to the manufacturer's protocol. 1 µg of RNA was reverse transcribed using Transcriptor First Strand cDNA Synthesis Kit (Roche). qPCR was performed with a Sybrgreen platform on a Bio-Rad CFX Cycler. Values were normalized to the expression of *Rpl32* as an internal control. For primer sequences see Table S5.

### Yeast two-hybrid assay

For yeast two-hybrid experiments the Matchmaker GAL4 Two-Hybrid System 3 from Clontech was used. To generate yeast constructs the ORFs were PCR amplified using specific primers with linked restriction sites (Table S6). To amplify *tbrd-1*, *tbrd-3* and *nht* genomic DNA was used. To exclude introns, cDNA was used to amplify *tbrd-2*, *sa*, *can*, *mia* and *rye*. cDNA was synthesized using innuScript Reverse Transcriptase (analytik jena) or iScript cDNA Synthesis Kit (BIO-RAD). The PCR fragments were inserted into pCRII-TOPO Vector (Invitrogen). Subsequently, the ORFs were ligated in frame into *pGBKT7* (*bait* vector) and *pGADT7* (*prey* vector) that were opened with the appropriate restriction enzymes. Yeast two-hybrid experiments were performed according to the manufacturer's manual.

### Co-immunoprecipitation

Co-IPs were carried out using the GFP-Trap A Kit (ChromoTek). Protein extracts were made from 200 adult testes (in case of *tbrd-1-eGFP*, *wild-type* and *tbrd-1* mutants) or 400 adult testes (in case of *tbrd-2-eGFP*). Testes were homogenized in 200 µl lysis buffer (supplied with the GFP-Trap A Kit) that include 2 µl 100× protease inhibitor (Thermo Scientific). Protein extracts were recovered following the manufacturer's advice. After adding 500 µl dilution buffer 4% (*tbrd-1-eGFP* and *wild-type*) or 8% (*tbrd-2-eGFP*) of the extract was retained as the input control. The remaining extract was incubated with 30 µl of GFP trap-A beads for 2.5 h at 4°C. Beads were washed twice and bound proteins were eluted by boiling in 100 µl SDS sample buffer for 10 min at 95°C. The whole supernatant was applied to a 10% SDS-gel. Western blots were performed using standard methods. Anti-tBRD-1 [19] was used at 1∶1000 in 5% dry milk in TBST. Anti-GFP (polyclonal rabbit antiserum was raised against full length GFP; unpublished; kindly provided by Joerg Leers) was used at a 1∶2000 dilution in 5% dry milk in TBST. POD-conjugated anti-rabbit antibody was subsequently applied at 1∶5000 (Jackson Immunology). Novex ECL Chemiluminescent Substrate Reagent Kit (Invitrogen) was used according to the manufacturer's recommendation.

### Immunofluorescence staining

Hoechst staining was used to visualize chromatin. All antibodies were used in immunofluorescence stainings of squashed testis carried out essentially as described before [51] and [52], [53]. We raised a peptide antibody (aa 175 to aa 189) against tBRD-3 in rabbit. The peptide sequence showed no sequence homology to tBRD-1 or tBRD-2. The affinity-purified antibody was applied in a dilution 1∶1000 (Pineda-Antibody-Service; http://www.pineda-abservice.de). Anti-tBRD-1 was used in a dilution 1∶5000 [19]. For peptide-blocking experiments the anti-tBRD-3 and anti-tBRD-1 antibodies were incubated with different concentrations of the tBRD-3 peptide (2–20 µg/ml) before carrying out the immunofluorescence stainings. To analyze the acetylation level after inhibitor treatment an anti-histone H4 acetyl-antibody (Millipore 06-598; 1∶200) was used. Cy3-conjugated anti-rabbit (Dianova; 1∶100) and Cy5-conjugated anti-rabbit (Dianova; 1∶100) were used as secondary antibodies. Immunofluorescence, eGFP and Hoechst signals were examined using a Zeiss microscope (AxioPlan2) equipped with appropriate fluorescence filters. Images were individually recorded and processed with Adobe Photoshop 7.0.

### Culture of pupal testis and inhibitor treatment

Pupal testis were dissected, cultured and treated with inhibitors as previously described [31], [19] and [32].

## Supporting Information

Figure S1 **tBRD-1 is required for gene repression in the testis.** (A) Gene expression was measured in wild-type and *tbrd-1^1^* mutant testis by Affymetrix microarrays in 5 replicates. Differentially expressed genes were identified after normalization RMA [54] using limma [55]. Of the genes with a p-value ≤0.05 (after Benjamini-Hochberg correction [56]) and an absolute log2-fold change ≥1, 419 genes were down-regulated and 223 were up-regulated. (B) Quantitative real-time PCR (qPCR) using cDNA of wild-type, *tbrd-1^1^* and *tbrd-1-eGFP*; *tbrd-1^1^* testes. Transcript levels of *CG31750*, *cutlet*, *twdlV* and *CG1441* were enriched in *tbrd-1* mutant testes compared to wild-type and *tbrd-1-eGFP*; *tbrd-1^1^* testes. P-values for significance: ** p≤0.01 and *** p≤0.001.
(TIF)

Figure S2 **tBRD-2 and tBRD-3 are predominantly transcribed in the testis.** (A) The *tbrd-2*-specific primers amplified a 319 bp cDNA fragment from the open reading frame of *tbrd-2* in testes (t) but not in carcass males (c) or in adult females (f). Additionally, in testes (t), in carcass males (c) and in adult females (f) a 373 bp fragment due to DNA contamination was amplified. (B) The *tbrd-3*-specific primers amplified a 462 bp cDNA fragment from the open reading frame of *tbrd-3* in testes (t) and in larvae (l). (A′,B′) A 372 bp cDNA fragment of the *β3-tubulin* gene amplified as a control was visible in all samples. Total RNA was used in A and A′, polyA^+^-mRNA was used in B and B′. +RT: with reverse transcriptase. −RT: without reverse transcriptase.
(TIF)

Figure S3 **The tBRD-3 peptide specifically blocks the anti-tBRD-3 antibody.** Single primary spermatocytes from wild-type testis stained with anti-tBRD-3 antibody (A), peptide-neutralized anti-tBRD-3 antibody (B), anti-tBRD-1 antibody (C) or anti-tBRD-1 antibody pre-incubated with the tBRD-3 peptide (D). tBRD-3 was no longer detectable with peptide-neutralized anti-tBRD-3 antibody (B) whereas blocking the tBRD-1 antibody with the tBRD-3 peptide did not affect the detection of tBRD-1 (D). (A′,B′,C′,D′) Hoechst DNA staining. (A″,B″,C″,D″) Phase-contrast images. Scale bars: 5 µm.
(TIF)

Figure S4 **Recruitment of tBRD-2 to the chromosomes is independent of the tTAF Sa.** Single primary spermatocytes from heterozygous (A panels) and homozygous *sa^2^* (B panels) mutants that express tBRD-2-eGFP stained with anti-tBRD-1 antibody. (A″,B″) In both heterozygous and homozygous *sa^2^* mutant spermatocytes tBRD-2-eGFP partially co-localized with tBRD-1 over the chromosomes (arrows). (A′″,B′″) Hoechst DNA staining. (A″″,B″″) Phase-contrast images. Scale bars: 5 µm.
(TIF)

Figure S5 **Localization of tBRD-1-eGFP and tBRD-2-eGFP is acetylation dependant.** Pupal testis of tBRD-1-eGFP (A-C″) or tBRD-2-eGFP (D–F″) expressing flies were treated with TSA or anacardic acid (AA) for 24 hours in culture and afterwards spermatoyctes were stained with an antibody against acetylated histone H4 (H4ac) (A′,B′,C′,D′,E′,F′). (A and D panels) Untreated control. (A,B,C,D,E,F) Hoechst DNA staining. (B and E panels) Incubation of testis with TSA led to increased histone H4 acetylation (B′,E′) and increased localization of tBRD-1-eGFP (B″) and tBRD-2-eGFP (E″) to the chromosomes (arrowheads) in comparison to the control (A″,D″). (C and F panels) Incubation of testis with AA led to a decrease in histone H4 acetylation (C′,F′) and altered localization of tBRD-1-eGFP (C″) and tBRD-2-eGFP (F″) to the chromosome territories (arrowheads). Scale bars: 20 µm in A–C″, 5 µm in D–F″.
(TIF)

Figure S6 **Co-localization of tBRD-1-eGFP and tBRD-3 is acetylation dependant.** Pupal testis of tBRD-1-eGFP expressing flies were treated with anacardic acid (AA) (B panels) or TSA (D panels) for 24 hours in culture and afterwards spermatoyctes were stained with an antibody against tBRD-3. (A and C panels) Untreated control. (B′,B″) Incubation of testis with AA led to a loss of tBRD-3 localization to the chromosome territories and co-localization between tBRD-3 and tBRD-1-eGFP was no longer detectable (arrows). (D′) TSA treatment led to increased localization of tBRD-3 to the chromosomes (arrow) in comparison to the control (C′). (D″) Partial co-localization of tBRD-1-eGFP and tBRD-3 was not affected by TSA treatment. (A′″, B′″, C ′″,D′″) Hoechst DNA staining. (A″″,B″″,C″″,D″″) Phase-contrast images. Scale bars: 5 µm.
(TIF)

Figure S7 **Overview of yeast two-hybrid experiments.** Positive (DBD-53+AD-T) and negative (DBD-Lam+AD-T) controls are shown on each plate. (A) Interaction of tBRD-1 and tBRD-3. tBRD-1 and tBRD-3 fusion proteins showed no self-activity. No homodimerization of tBRD-3 was detectable. (B) Interaction of tBRD-2 and tBRD-3. tBRD-2 and tBRD-3 fusion proteins showed no self-activity. (C) Interaction of tBRD-1 and tBRD-2. tBRD-2 was not able to interact with itself, tBRD-3 or Sa. tBRD-3 showed no interaction with Sa. (D) Interaction of tBRD-1 and Rye. tBRD-1 and Rye fusion proteins showed no self-activity. (E) Interaction of tBRD-1 and Can when Can is acting as the bait. Both tBRD-1 fusion proteins and AD-Can showed no self-activity. Weak self-activity was detectable for DBD-Can. Nevertheless, a clear difference between the self-activity of DBD-Can compared to DBD-Can+AD-tBRD-1 was visible. (F) tBRD-3 and Sa could interact when tBRD-3 acts as the bait. tBRD-3 and Sa fusion proteins showed no self-activity. (G) Interaction of tBRD-1 and Can when Can acts as the bait. Can was not able to interact with tBRD-2 or tBRD-3. A few blue colonies were detectable for DBD-Can+AD-tBRD-2 and DBD-Can+AD-tBRD-3 and resulted from the self-activity of DBD-Can (shown on plate E). (H) Interaction of Rye with tBRD-1, tBRD-2 and tBRD-3. Rye was not able to interact with Sa. (I) Nht showed no interaction with tBRD-1, tBRD-2 or tBRD-3. (J) Mia showed no interaction with tBRD-1, tBRD-2 or tBRD-3. However, a control that shows expression of Mia fusion proteins in yeast is missing. (K) Homodimerization of Rye. Mia, Sa and Nht were not able to form homodimers. Blue colonies were detectable for DBD-Can+AD-Can that might result from the self-activity of DBD-Can. (L) Homodimerization of Rye. Mia, Sa and Nht were not able to form homodimers. Sa and Nht showed no interaction. (M) Mia showed no interaction with Sa, Rye or Nht. Mia fusion proteins showed no self-activity. As above, a control that shows expression of Mia fusion proteins in yeast is missing. (N) Interaction of Rye and Nht. Rye and Nht fusion proteins showed no self-activity. (O) Can was not able to interact with Sa, Nht or Rye. A few blue colonies were detectable that might result from the self-activity of DBD-Can (shown on plate E and P). (P) Weak self-activity was detectable for DBD-Can but not for AD-Can. Can was not able to interact with Rye or Mia. A few blue colonies were detectable that might result from the self-activity of DBD-Can.
(TIF)

Table S1 **Summary of self-activity tests of the different plasmids used for yeast two-hybrid experiments.**
(PDF)

Table S2 **Summary of yeast two-hybrid experiments for the three bromodomain proteins tBRD-1, tBRD-2 and tBRD-3.**
(PDF)

Table S3 **Summary of yeast two-hybrid experiments for tBRD-1, tBRD-2, tBRD-3 and tTAFs.**
(PDF)

Table S4 **Summary of yeast two-hybrid experiments for the five tTAFs.**
(PDF)

Table S5 **Primers used for qPCRs.**
(PDF)

Table S6 **Oligonucleotids used for yeast constructs.**
(PDF)

Dataset S1 (XLSX)
