# Supplementary figures and images for: tBRD-1 Selectively Controls Gene Activity in the Drosophila Testis and Interacts with Two New Members of the Bromodomain and Extra-Terminal (BET) Family
Source: PLoS One. 2014 Sep 24;9(9):e108267. doi: 10.1371/journal.pone.0108267 (PMC4177214; doi:10.1371/journal.pone.0108267)

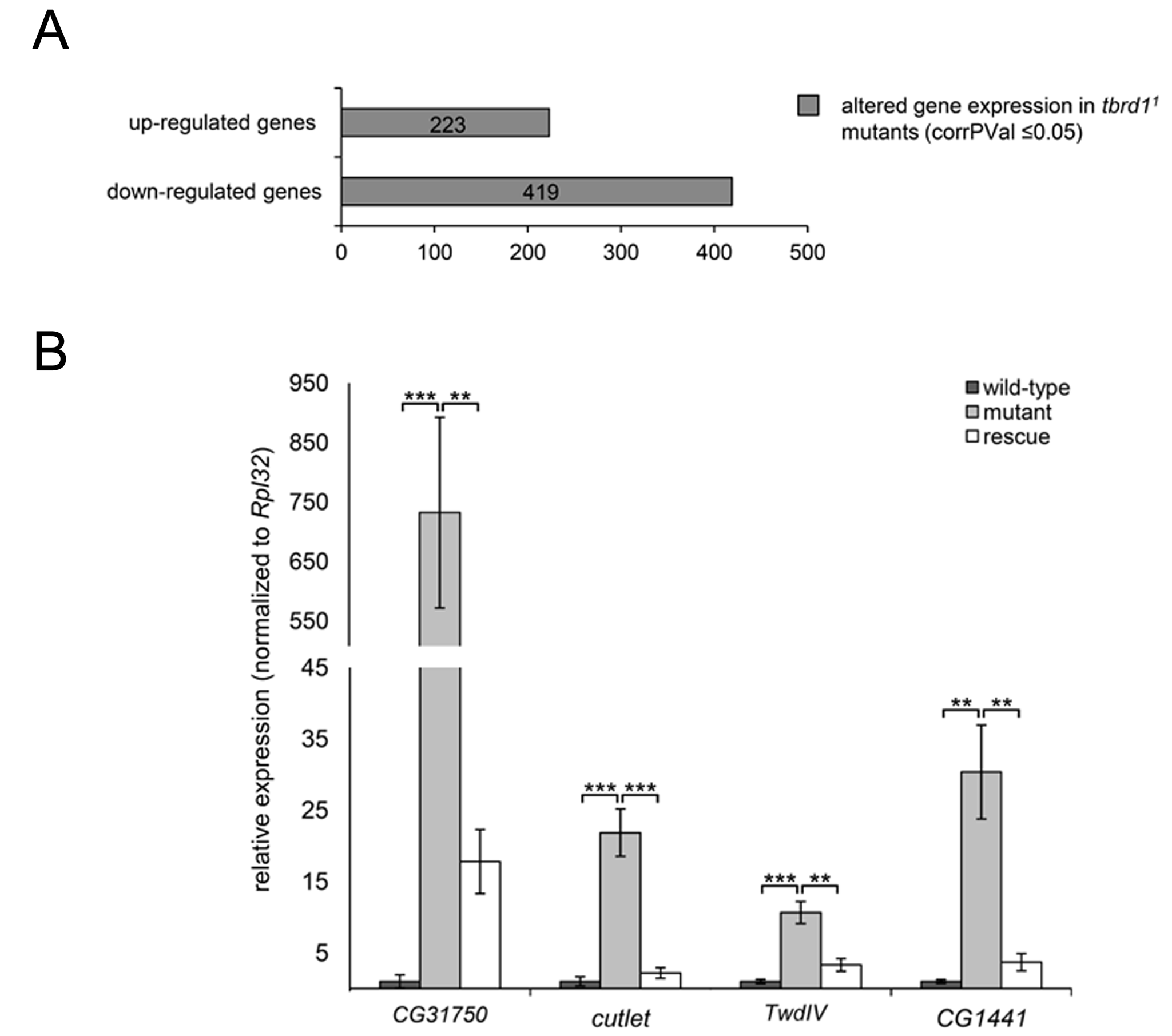

Supplement: Figure S1 — tBRD-1 is required for gene repression in the testis. (A) Gene expression was measured in wild-type and tbrd-11 mutant testis by Affymetrix microarrays in 5 replicates. Differentially expressed genes were identified after normalization RMA [54] using limma [55]. Of the genes with a p-value ≤0.05 (after Benjamini-Hochberg correction [56]) and an absolute log2-fold change ≥1, 419 genes were down-regulated and 223 were up-regulated. (B) Quantitative real-time PCR (qPCR) using cDNA of wild-type, tbrd-11 and tbrd-1-eGFP; tbrd-11 testes. Transcript levels of CG31750, cutlet, twdlV and CG1441 were enriched in tbrd-1 mutant testes compared to wild-type and tbrd-1-eGFP; tbrd-11 testes. P-values for significance: ** p≤0.01 and *** p≤0.001. (TIF) [file pone.0108267.s001.tif]

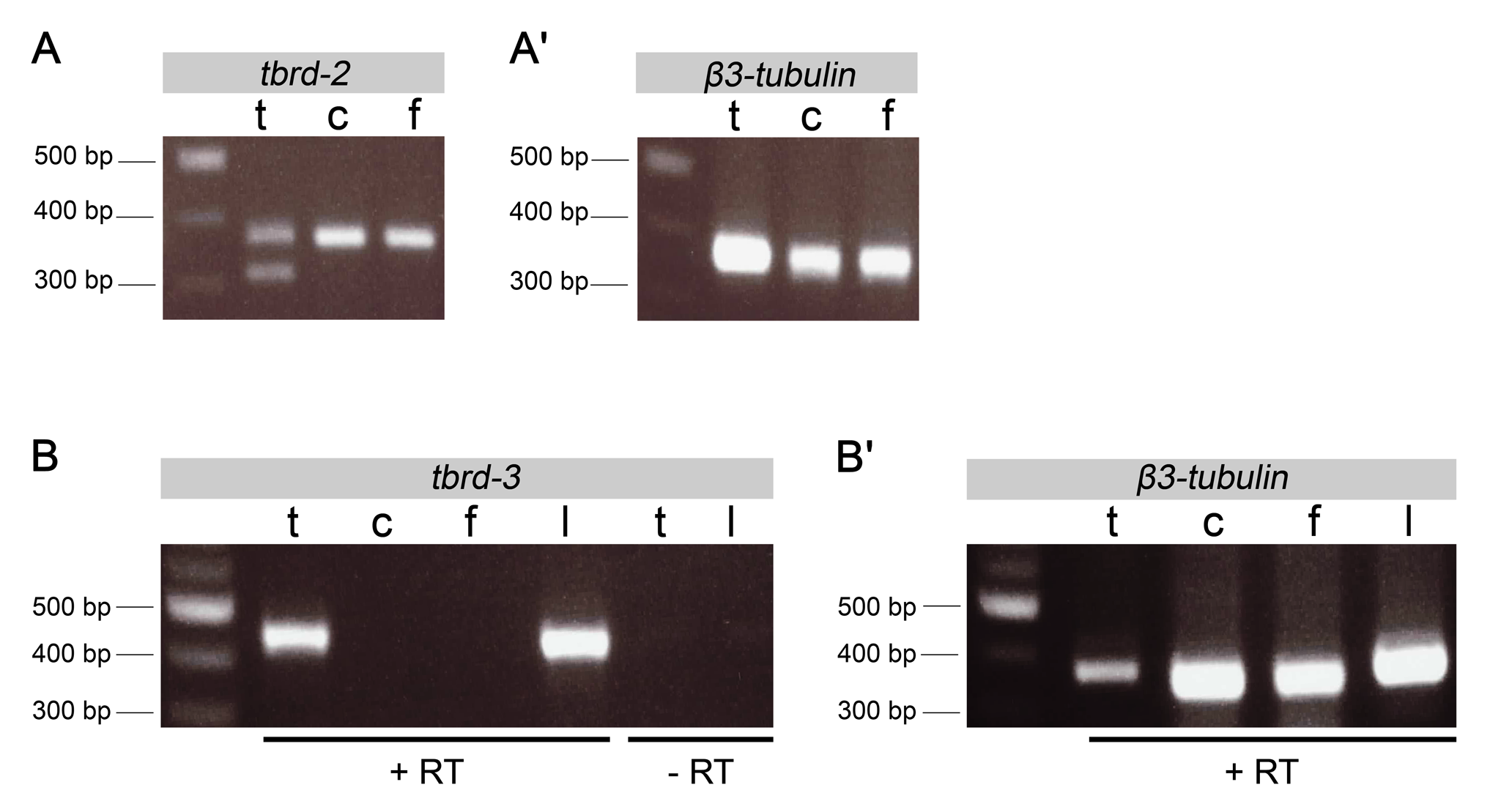

Supplement: Figure S2 — tBRD-2 and tBRD-3 are predominantly transcribed in the testis. (A) The tbrd-2-specific primers amplified a 319 bp cDNA fragment from the open reading frame of tbrd-2 in testes (t) but not in carcass males (c) or in adult females (f). Additionally, in testes (t), in carcass males (c) and in adult females (f) a 373 bp fragment due to DNA contamination was amplified. (B) The tbrd-3-specific primers amplified a 462 bp cDNA fragment from the open reading frame of tbrd-3 in testes (t) and in larvae (l). (A′,B′) A 372 bp cDNA fragment of the β3-tubulin gene amplified as a control was visible in all samples. Total RNA was used in A and A′, polyA+-mRNA was used in B and B′. +RT: with reverse transcriptase. −RT: without reverse transcriptase. (TIF) [file pone.0108267.s002.tif]

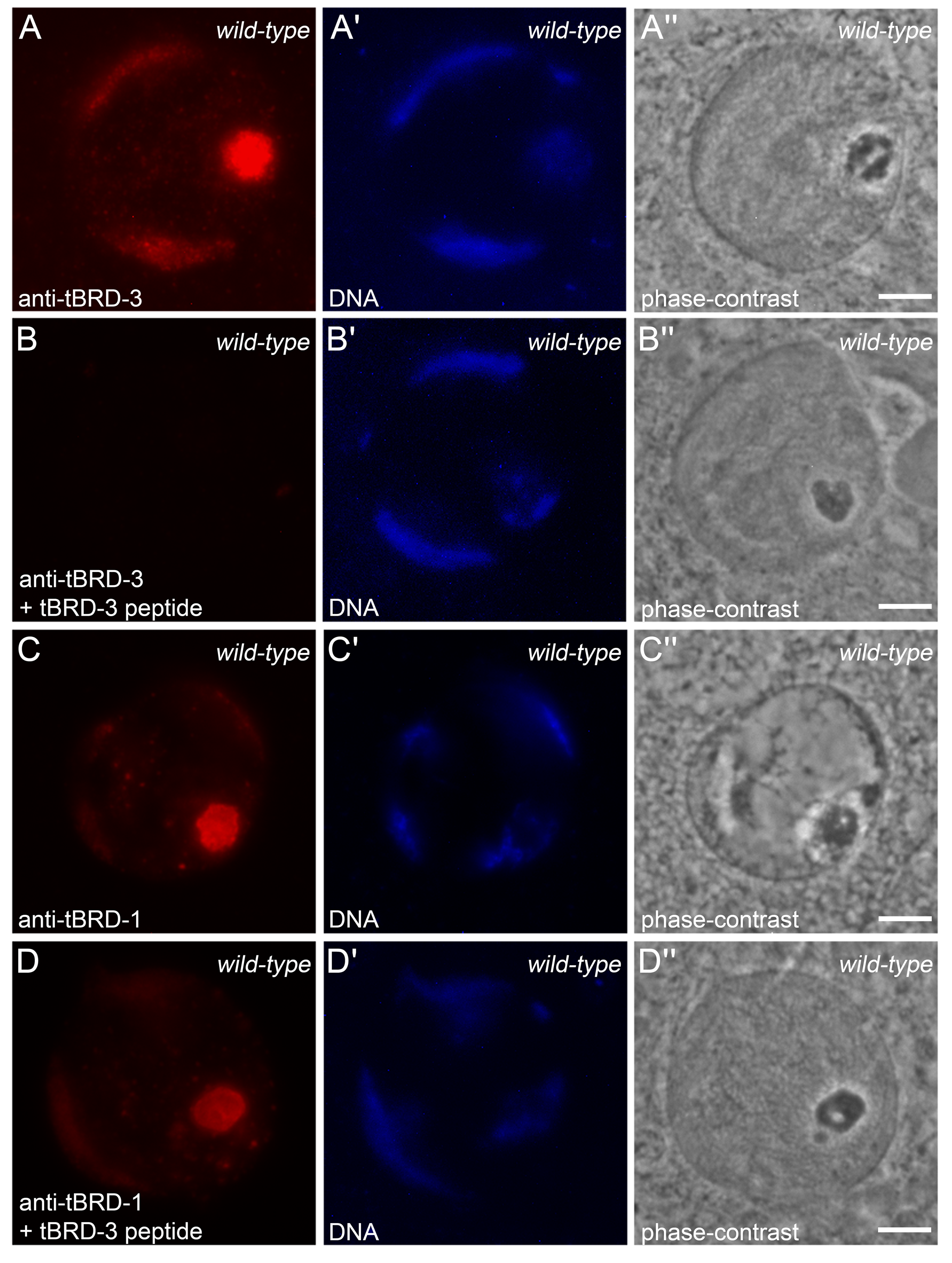

Supplement: Figure S3 — The tBRD-3 peptide specifically blocks the anti-tBRD-3 antibody. Single primary spermatocytes from wild-type testis stained with anti-tBRD-3 antibody (A), peptide-neutralized anti-tBRD-3 antibody (B), anti-tBRD-1 antibody (C) or anti-tBRD-1 antibody pre-incubated with the tBRD-3 peptide (D). tBRD-3 was no longer detectable with peptide-neutralized anti-tBRD-3 antibody (B) whereas blocking the tBRD-1 antibody with the tBRD-3 peptide did not affect the detection of tBRD-1 (D). (A′,B′,C′,D′) Hoechst DNA staining. (A″,B″,C″,D″) Phase-contrast images. Scale bars: 5 µm. (TIF) [file pone.0108267.s003.tif]

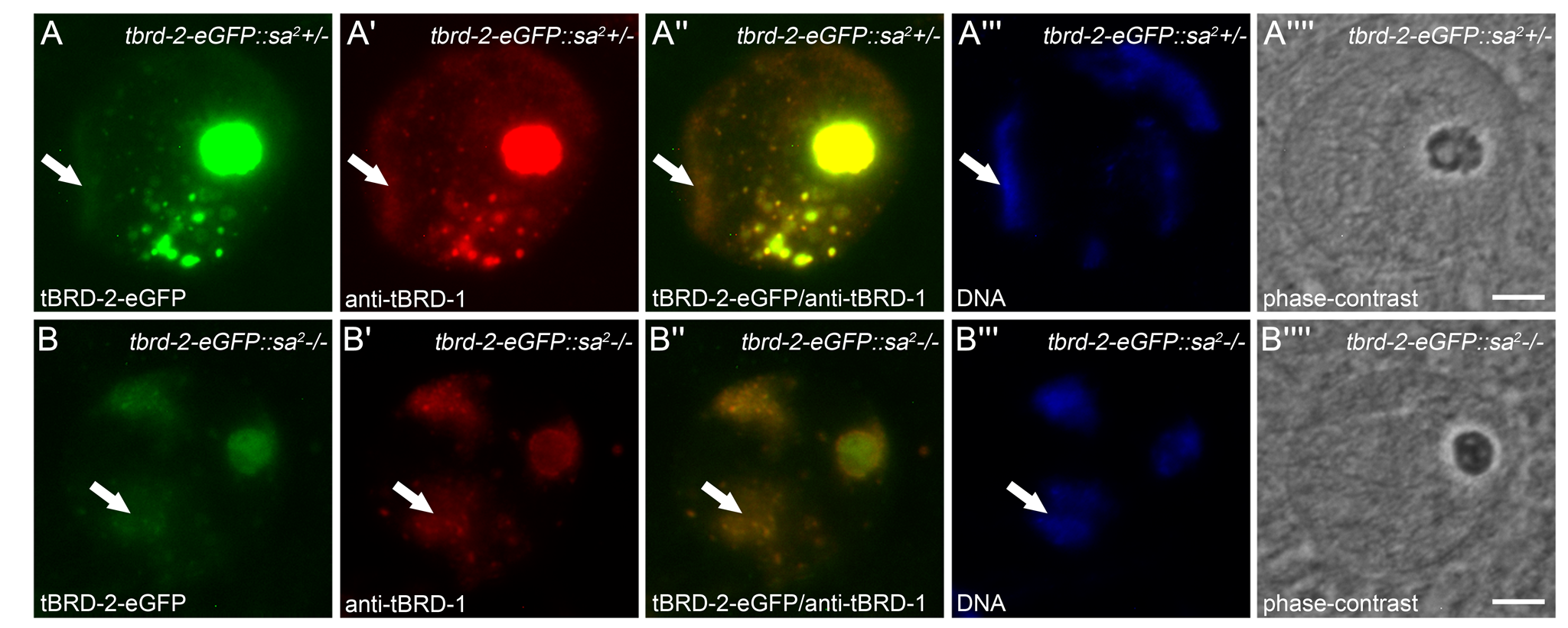

Supplement: Figure S4 — Recruitment of tBRD-2 to the chromosomes is independent of the tTAF Sa. Single primary spermatocytes from heterozygous (A panels) and homozygous sa2 (B panels) mutants that express tBRD-2-eGFP stained with anti-tBRD-1 antibody. (A″,B″) In both heterozygous and homozygous sa2 mutant spermatocytes tBRD-2-eGFP partially co-localized with tBRD-1 over the chromosomes (arrows). (A′″,B′″) Hoechst DNA staining. (A″″,B″″) Phase-contrast images. Scale bars: 5 µm. (TIF) [file pone.0108267.s004.tif]

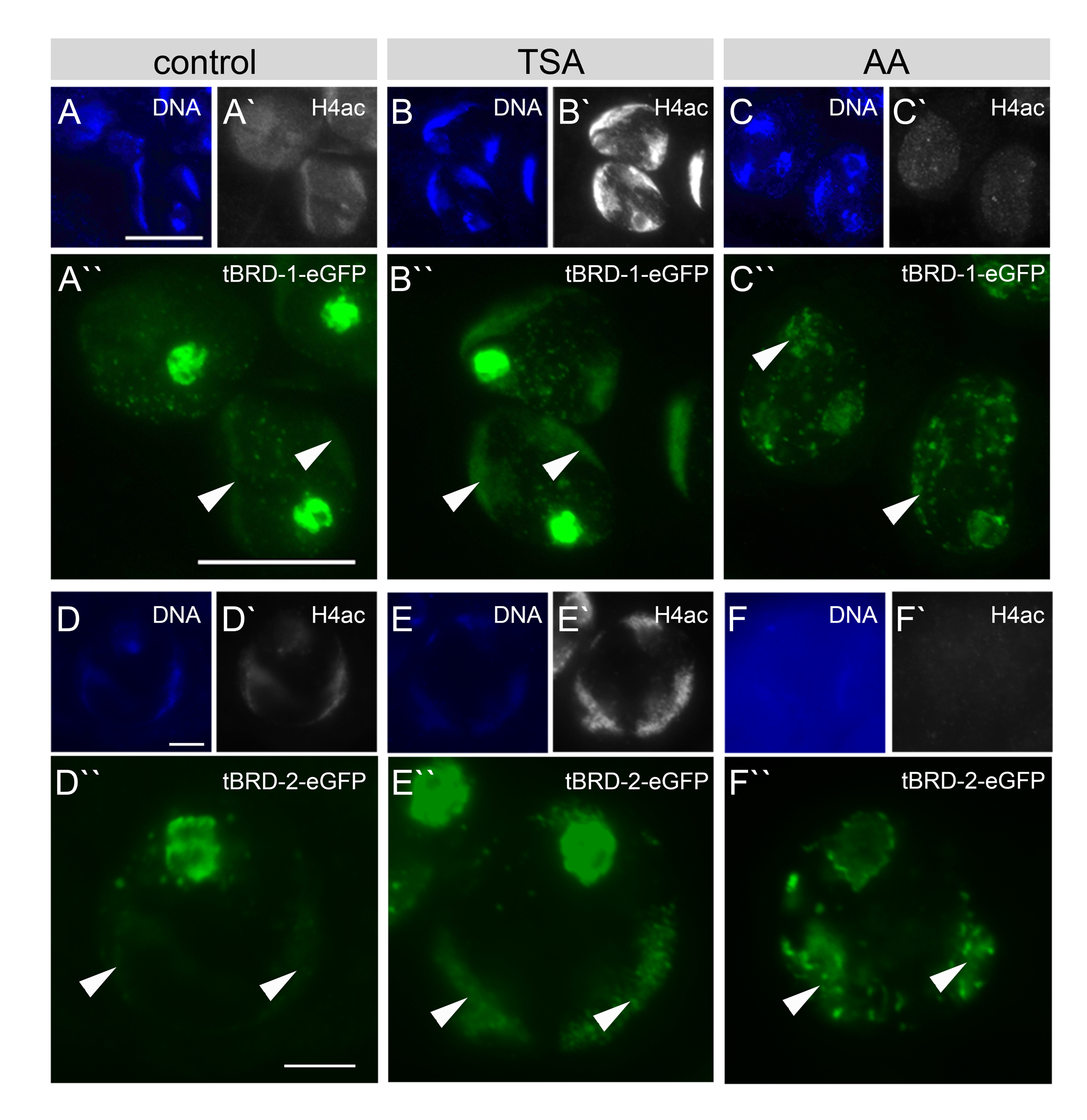

Supplement: Figure S5 — Localization of tBRD-1-eGFP and tBRD-2-eGFP is acetylation dependant. Pupal testis of tBRD-1-eGFP (A-C″) or tBRD-2-eGFP (D–F″) expressing flies were treated with TSA or anacardic acid (AA) for 24 hours in culture and afterwards spermatoyctes were stained with an antibody against acetylated histone H4 (H4ac) (A′,B′,C′,D′,E′,F′). (A and D panels) Untreated control. (A,B,C,D,E,F) Hoechst DNA staining. (B and E panels) Incubation of testis with TSA led to increased histone H4 acetylation (B′,E′) and increased localization of tBRD-1-eGFP (B″) and tBRD-2-eGFP (E″) to the chromosomes (arrowheads) in comparison to the control (A″,D″). (C and F panels) Incubation of testis with AA led to a decrease in histone H4 acetylation (C′,F′) and altered localization of tBRD-1-eGFP (C″) and tBRD-2-eGFP (F″) to the chromosome territories (arrowheads). Scale bars: 20 µm in A–C″, 5 µm in D–F″. (TIF) [file pone.0108267.s005.tif]

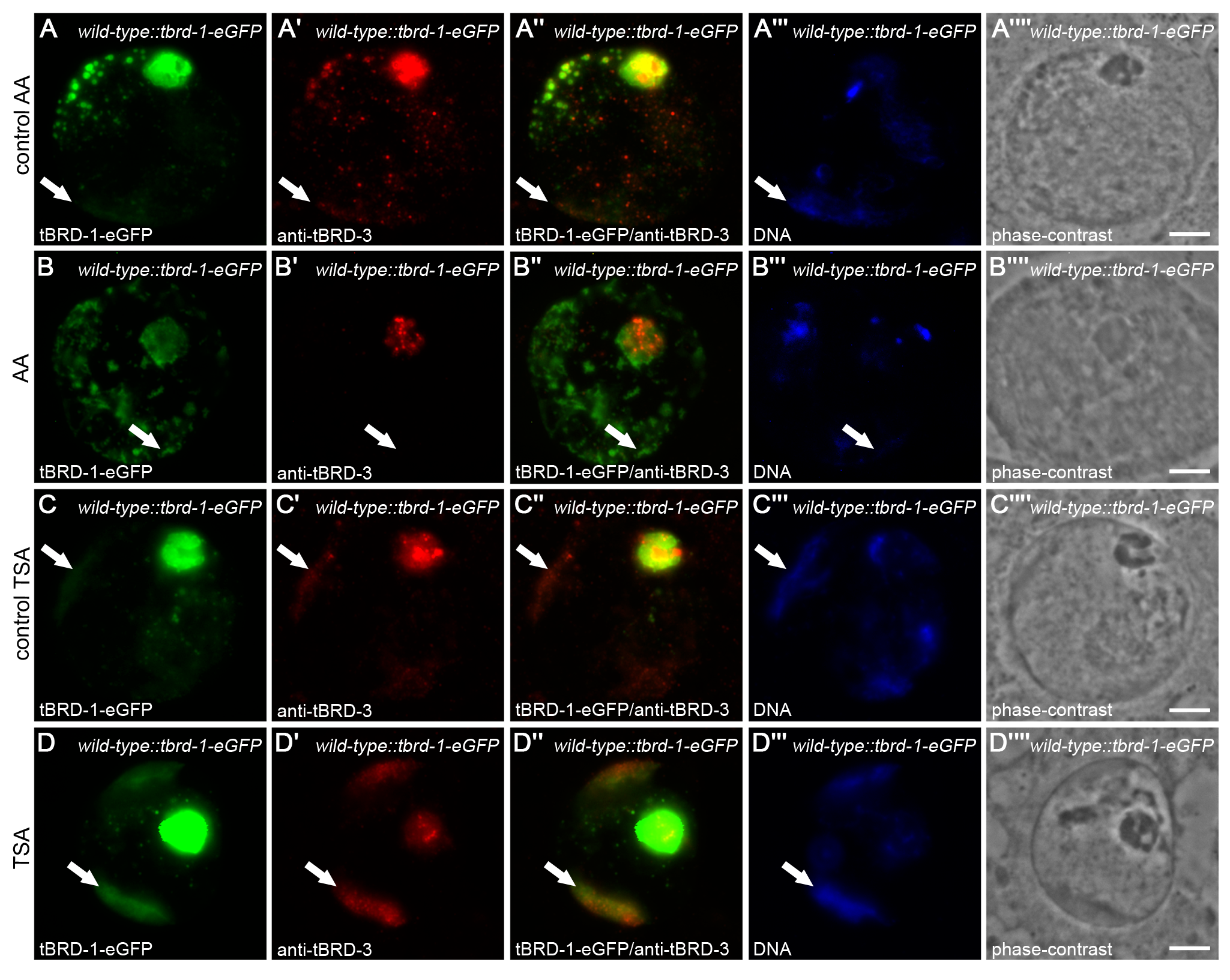

Supplement: Figure S6 — Co-localization of tBRD-1-eGFP and tBRD-3 is acetylation dependant. Pupal testis of tBRD-1-eGFP expressing flies were treated with anacardic acid (AA) (B panels) or TSA (D panels) for 24 hours in culture and afterwards spermatoyctes were stained with an antibody against tBRD-3. (A and C panels) Untreated control. (B′,B″) Incubation of testis with AA led to a loss of tBRD-3 localization to the chromosome territories and co-localization between tBRD-3 and tBRD-1-eGFP was no longer detectable (arrows). (D′) TSA treatment led to increased localization of tBRD-3 to the chromosomes (arrow) in comparison to the control (C′). (D″) Partial co-localization of tBRD-1-eGFP and tBRD-3 was not affected by TSA treatment. (A′″, B′″, C ′″,D′″) Hoechst DNA staining. (A″″,B″″,C″″,D″″) Phase-contrast images. Scale bars: 5 µm. (TIF) [file pone.0108267.s006.tif]

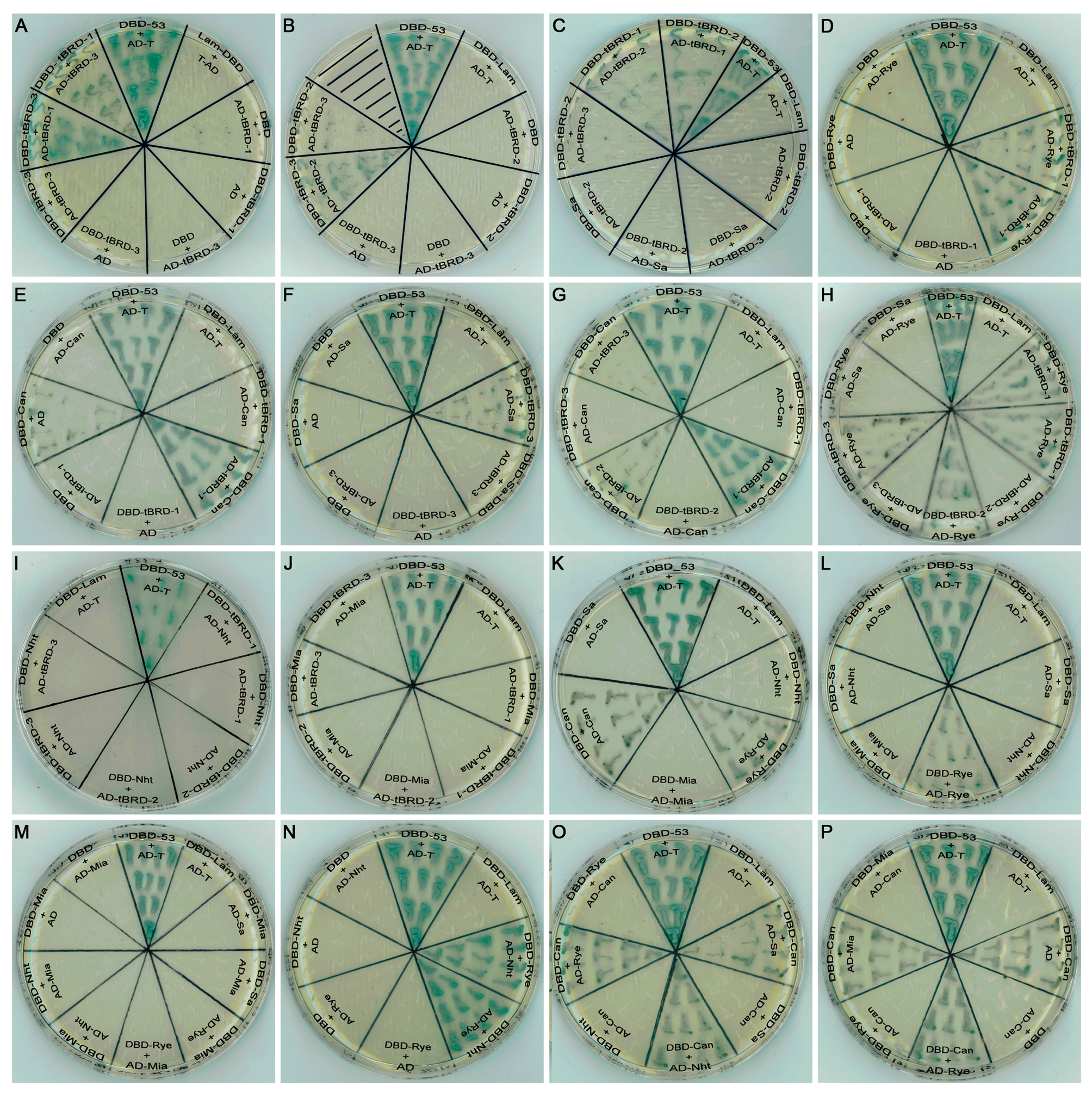

Supplement: Figure S7 — Overview of yeast two-hybrid experiments. Positive (DBD-53+AD-T) and negative (DBD-Lam+AD-T) controls are shown on each plate. (A) Interaction of tBRD-1 and tBRD-3. tBRD-1 and tBRD-3 fusion proteins showed no self-activity. No homodimerization of tBRD-3 was detectable. (B) Interaction of tBRD-2 and tBRD-3. tBRD-2 and tBRD-3 fusion proteins showed no self-activity. (C) Interaction of tBRD-1 and tBRD-2. tBRD-2 was not able to interact with itself, tBRD-3 or Sa. tBRD-3 showed no interaction with Sa. (D) Interaction of tBRD-1 and Rye. tBRD-1 and Rye fusion proteins showed no self-activity. (E) Interaction of tBRD-1 and Can when Can is acting as the bait. Both tBRD-1 fusion proteins and AD-Can showed no self-activity. Weak self-activity was detectable for DBD-Can. Nevertheless, a clear difference between the self-activity of DBD-Can compared to DBD-Can+AD-tBRD-1 was visible. (F) tBRD-3 and Sa could interact when tBRD-3 acts as the bait. tBRD-3 and Sa fusion proteins showed no self-activity. (G) Interaction of tBRD-1 and Can when Can acts as the bait. Can was not able to interact with tBRD-2 or tBRD-3. A few blue colonies were detectable for DBD-Can+AD-tBRD-2 and DBD-Can+AD-tBRD-3 and resulted from the self-activity of DBD-Can (shown on plate E). (H) Interaction of Rye with tBRD-1, tBRD-2 and tBRD-3. Rye was not able to interact with Sa. (I) Nht showed no interaction with tBRD-1, tBRD-2 or tBRD-3. (J) Mia showed no interaction with tBRD-1, tBRD-2 or tBRD-3. However, a control that shows expression of Mia fusion proteins in yeast is missing. (K) Homodimerization of Rye. Mia, Sa and Nht were not able to form homodimers. Blue colonies were detectable for DBD-Can+AD-Can that might result from the self-activity of DBD-Can. (L) Homodimerization of Rye. Mia, Sa and Nht were not able to form homodimers. Sa and Nht showed no interaction. (M) Mia showed no interaction with Sa, Rye or Nht. Mia fusion proteins showed no self-activity. As above, a control that shows expression of Mia [file pone.0108267.s007.tif]
