# Supplementary material for: tBRD-1 Selectively Controls Gene Activity in the Drosophila Testis and Interacts with Two New Members of the Bromodomain and Extra-Terminal (BET) Family
Source: PLoS One. 2014 Sep 24;9(9):e108267. doi: 10.1371/journal.pone.0108267 (PMC4177214; doi:10.1371/journal.pone.0108267)
Supplement: Table S1 — Summary of self-activity tests of the different plasmids used for yeast two-hybrid experiments. (PDF) [file pone.0108267.s008.pdf]

Table S1. Summary of self-activity tests of the different plasmids used for yeast two-hybrid experiments.

Bait (pGBKT7)	Prey (pGADT7)	Growth and blue color
tBRD-1	AD	-
DBD	tBRD-1	-
tBRD-2	AD	-
DBD	tBRD-2	-
tBRD-3	AD	-
DBD	tBRD-3	-
Spermatocyte arrest	AD	-
DBD	Spermatocyte arrest	-
Cannonball	AD	+
DBD	Cannonball	-
Meiotic arrest	AD	-
DBD	Meiotic arrest	-
Ryan express	AD	-
DBD	Ryan express	-
No hitter	AD	-
DBD	No hitter	-
		- no<br>+ weak<br>++ intermediate<br>+++ strong
