# Supplementary material for: tBRD-1 Selectively Controls Gene Activity in the Drosophila Testis and Interacts with Two New Members of the Bromodomain and Extra-Terminal (BET) Family
Source: PLoS One. 2014 Sep 24;9(9):e108267. doi: 10.1371/journal.pone.0108267 (PMC4177214; doi:10.1371/journal.pone.0108267)
Supplement: Table S2 — Summary of yeast two-hybrid experiments for the three bromodomain proteins tBRD-1, tBRD-2 and tBRD-3. (PDF) [file pone.0108267.s009.pdf]

Table S2. Summary of yeast two-hybrid experiments for the three bromodomain proteins tBRD-1, tBRD-2 and tBRD-3.

Bait (pGBKT7)	Prey (pGADT7)	Growth and blue color
tBRD-1	tBRD-1	+++
tBRD-2	tBRD-2	-
tBRD-3	tBRD-3	-
tBRD-1	tBRD-2	+++
tBRD-2	tBRD-1	+++
tBRD-1	tBRD-3	++
tBRD-3	tBRD-1	+++
tBRD-2	tBRD-3	+
tBRD-3	tBRD-2	++

- no
+ weak
++ intermediate
+++ strong
