# Supplementary material for: tBRD-1 Selectively Controls Gene Activity in the Drosophila Testis and Interacts with Two New Members of the Bromodomain and Extra-Terminal (BET) Family
Source: PLoS One. 2014 Sep 24;9(9):e108267. doi: 10.1371/journal.pone.0108267 (PMC4177214; doi:10.1371/journal.pone.0108267)
Supplement: Table S3 — Summary of yeast two-hybrid experiments for tBRD-1, tBRD-2, tBRD-3 and tTAFs. (PDF) [file pone.0108267.s010.pdf]

Table S3. Summary of yeast two-hybrid experiments for tBRD-1, tBRD-2, tBRD-3 and tTAFs.

Bait (pGBKT7)	Prey (pGADT7)	Growth and blue color
tBRD-1	Spermatocyte arrest	-
Spermatocyte arrest	tBRD-1	+++
tBRD-1	Cannonball	-
Cannonball	tBRD-1	+++*
tBRD-1	Meiotic arrest	-
Meiotic arrest	tBRD-1	-
tBRD-1	Ryan express	++
Ryan express	tBRD-1	++
tBRD-1	No hitter	-
No hitter	tBRD-1	-
tBRD-2	Spermatocyte arrest	-
Spermatocyte arrest	tBRD-2	-
tBRD-2	Cannonball	-
Cannonball	tBRD-2	+*
tBRD-2	Meiotic arrest	-
Meiotic arrest	tBRD-2	-
tBRD-2	Ryan express	++
Ryan express	tBRD-2	-
tBRD-2	No hitter	-
No hitter	tBRD-2	-
tBRD-3	Spermatocyte arrest	+
Spermatocyte arrest	tBRD-3	-
tBRD-3	Cannonball	-
Cannonball	tBRD-3	+*
tBRD-3	Meiotic arrest	-
Meiotic arrest	tBRD-3	-
tBRD-3	Ryan express	++
Ryan express	tBRD-3	-
tBRD-3	No hitter	-
No hitter	tBRD-3	-
* self-activity of DBD-Can		- no<br>+ weak<br>++ intermediate<br>+++ strong
