# Supplementary material for: tBRD-1 Selectively Controls Gene Activity in the Drosophila Testis and Interacts with Two New Members of the Bromodomain and Extra-Terminal (BET) Family
Source: PLoS One. 2014 Sep 24;9(9):e108267. doi: 10.1371/journal.pone.0108267 (PMC4177214; doi:10.1371/journal.pone.0108267)
Supplement: Table S4 — Summary of yeast two-hybrid experiments for the five tTAFs. (PDF) [file pone.0108267.s011.pdf]

Table S4. Summary of yeast two-hybrid experiments for the five tTAFs.

Bait (pGBKT7)	Prey (pGADT7)	Growth and blue color
Spermatocyte arrest	Spermatocyte arrest	-
Cannonball	Cannonball	+*
Meiotic arrest	Meiotic arrest	-
Ryan express	Ryan express	+
No hitter	No hitter	-
Spermatocyte arrest	Cannonball	-
Cannonball	Spermatocyte arrest	+*
Spermatocyte arrest	Meiotic arrest	-
Meiotic arrest	Spermatocyte arrest	-
Spermatocyte arrest	Ryan express	-
Ryan express	Spermatocyte arrest	-
Spermatocyte arrest	No hitter	-
No hitter	Spermatocyte arrest	-
Cannonball	Meiotic arrest	+*
Meiotic arrest	Cannonball	-
Cannonball	Ryan express	+*
Ryan express	Cannonball	-
Cannonball	No hitter	+*
No hitter	Cannonball	-
Meiotic arrest	Ryan express	-
Ryan express	Meiotic arrest	-
Meiotic arrest	No hitter	-
No hitter	Meiotic arrest	-
Ryan express	No hitter	+++[1]
No hitter	Ryan express	+++[1]

*** self-activity**
[1] Interaction showed in [9]

- no
+ weak
++ intermediate
+++ strong
