# Supplementary material for: tBRD-1 Selectively Controls Gene Activity in the Drosophila Testis and Interacts with Two New Members of the Bromodomain and Extra-Terminal (BET) Family
Source: PLoS One. 2014 Sep 24;9(9):e108267. doi: 10.1371/journal.pone.0108267 (PMC4177214; doi:10.1371/journal.pone.0108267)
Supplement: Table S5 — Primers used for qPCRs. (PDF) [file pone.0108267.s012.pdf]

Table S5. Primers used for qPCRs.

rpl32-fw	ATGACCATCCGCCCAGCATAC
rpl32-rev	CTGCATGAGCAGGACCTCCAG
CG18673-fw	ATATGAGACGATGGGCGAGG
CG18673-rev	GGGAAACACAGTCCAGGTGA
CG13946-fw	GGAGGACCTCAAAATGGTCA
CG13946-rev	GCCTCAGTGGTCGAAGTTGT
CG32457-fw	AATTGGGTTGGCATTGGTAA
CG32457-rev	CCTGATTGTCACAGGTGGTC
CG17917-fw	CACATCGGACCGCAGGAG
CG17917-rev	AGGAAGACGAACCGATGGGT
yp3-fw	CACCTGGGAGCGTTTGGAG
yp3-rev	GCTGAGCGGGGCTGGTCT
CG42827-fw	ATGCGTTTGACAATCTTATGTAT
CG42827-rev	CACGCAACTTTCCTTGGTA
CG42828-fw	GCTTACCCAGTTTTTGTGC
CG42828-rev	TAATTAGCGAGAACAAATCGG
tfIIa-s-fw	TTAAGGCAGCCACTATGTCGT
tfIIa-s-rev	CTTGTCGAATTGCAGCAGAA
gr36d-fw	CCCCTTTGTTGAGGATGCTA
gr36d-rev	TGATTCCTAATGGGTTGTGC
cutlet-fw	GAGAATGGCACGCAAATGT
cutlet-rev	AGGTCGCTTCAAGACGTTGT
twdIV-fw	CACCCCAGGGCTACAACTAT
twdIV-rev	CGGCAGGCTGTTGGTAGA
CG1441-fw	CATTTATCTGCTCATCCGCCC
CG1441-rev	GAACACGGCATTTCGTAGGG
