# Supplementary material for: tBRD-1 Selectively Controls Gene Activity in the Drosophila Testis and Interacts with Two New Members of the Bromodomain and Extra-Terminal (BET) Family
Source: PLoS One. 2014 Sep 24;9(9):e108267. doi: 10.1371/journal.pone.0108267 (PMC4177214; doi:10.1371/journal.pone.0108267)
Supplement: Table S6 — Oligonucleotids used for yeast constructs. (PDF) [file pone.0108267.s013.pdf]

Table S6. Oligonucleotids used for yeast constructs.

tBRD1-Y2H-NdeI-fw	CATATGATGAATGAACTGCAGTCGAAT
tBRD1-Y2H-EcoRI-rev	GAATTCTTAATCGCTATCATAAGTTTGGT
tBrd2-Y2H-NdeI-fw	CATATGATGGCATCTTGCAAGCCG
tBrd2-Y2H-EcoRI-rev	GAATTCTTAGGCCCTCAGCTGTC
tBrd3-Y2H-NdeI-fw	CATATGATGGCTGCAAAACAAGAAAC
tBrd3-Y2H-EcoRI-rev	GAATTCTTAATTATATTTGGGCTTGGTG
Sa-Y2H-EcoRI-fw	CATATGATGAATACCTACGACGAAGTC
Sa-Y2H-BamHI-rev	GGATCCTTAATTCGGTTTTTCAATACCTA
Nh-Y2H-EcoRI-fw	GAATTCATGTCAATTATTTCCCTTG
Nh-Y2H-BamHI-rev	GGATCCTTATGACTTGTATTTTAAATA
Can-Y2H-NdeI-fw	CATATGGATTTACCATTGCCAATAC
Can-Y2H-BamHI-rev	GGATCCTTAAGATTTGTTCTCCTT
Mia-Y2H-NdeI-fw	CATATGTGGCAGTTTGAAAACAAAA
Mia-Y2H-BamHI-rev	GGATCCTTAAGTAATTATTGGACAA
Rye-Y2H-NdeI-fw	CATATGGATAGTAACATGGAACC
Rye-Y2H-BamHI-rev	GGATCCTTATTCAATTGGGAACT
